# miRNA-211 maintains metabolic homeostasis in medulloblastoma through its target gene long-chain acyl-CoA synthetase 4

**DOI:** 10.1186/s40478-023-01684-w

**Published:** 2023-12-19

**Authors:** Menglang Yuan, Iqbal Mahmud, Keisuke Katsushima, Kandarp Joshi, Olivier Saulnier, Rudramani Pokhrel, Bongyong Lee, Wathsala Liyanage, Haritha Kunhiraman, Stacie Stapleton, Ignacio Gonzalez-Gomez, Rangaramanujam M. Kannan, Tanja Eisemann, Elayaraja Kolanthai, Sudipta Seal, Timothy J. Garrett, Saed Abbasi, Kimberly Bockley, Justin Hanes, Prem Chapagain, George Jallo, Robert J. Wechsler-Reya, Michael D. Taylor, Charles G. Eberhart, Animesh Ray, Ranjan J. Perera

**Affiliations:** 1 Department of Oncology, Sidney Kimmel Comprehensive Cancer Center, School of Medicine, Johns Hopkins University, 1650 Orleans St., Baltimore, MD 21231 USA; 2 Johns Hopkins All Children’s Hospital, 600 5th St. South, St. Petersburg, FL 33701 USA; 3 Department of Bioinformatics and Computational Biology, The University of Texas MD Anderson Cancer Center, Houston, TX 77030 USA; 4 The Arthur and Sonia Labatt Brain Tumour Research Centre and the Developmental and Stem Cell Biology Program, The Hospital for Sick Children, Toronto, ON Canada; 5 Center for Nanomedicine at the Wilmer Eye Institute, Johns Hopkins University School of Medicine, Baltimore, MD 21231 USA; 6 Department of Chemical and Biomolecular Engineering, Johns Hopkins University, Baltimore, MD 21218 USA; 7 National Cancer Institute-Designated Cancer Center, Sanford Burnham Prebys Medical Discovery Institute, La Jolla, CA 92037 USA; 8 Advanced Materials Processing and Analysis Centre, Nanoscience Technology Center, Materials Science and Engineering, College of Medicine, University of Central Florida, Orlando, FL 32826 USA; 9 Department Pathology, Immunology and Laboratory Medicine, College of Medicine, University of Florida, Gainesville, FL 32610 USA; 10 Department of Ophthalmology, Johns Hopkins University School of Medicine, Baltimore, MD 21231 USA; 11 Department of Pharmacology and Molecular Sciences, Johns Hopkins University, Baltimore, MD 21205 USA; 12 Department of Physics, Florida International University, Miami, FL 33199 USA; 13 Herbert Irving Comprehensive Cancer Center, Columbia University Medical Center, New York, NY 10032 USA; 14 Texas Children’s Cancer Center, Hematology-Oncology Section, Houston, TX 77030 USA; 15 Department of Pediatrics—Hematology/Oncology and Neurosurgery, Baylor College of Medicine, Houston, TX 77030 USA; 16 Department of Pathology, Johns Hopkins University School of Medicine, Baltimore, MD 21205 USA; 17 Riggs School of Applied Life Sciences, Keck Graduate Institute, Claremont, CA 91711 USA; 18 Division of Biology and Biological Engineering, California Institute of Technology, Pasadena, CA 91125 USA

**Keywords:** Medulloblastoma, miRNA-211, Metabolism, Nanoparticles, Therapeutics

## Abstract

The prognosis of childhood medulloblastoma (MB) is often poor, and it usually requires aggressive therapy that adversely affects quality of life. microRNA-211 (miR-211) was previously identified as an important regulator of cells that descend from neural cells. Since medulloblastomas primarily affect cells with similar ontogeny, we investigated the role and mechanism of miR-211 in MB. Here we showed that miR-211 expression was highly downregulated in cell lines, PDXs, and clinical samples of different MB subgroups (SHH, Group 3, and Group 4) compared to normal cerebellum. miR-211 gene was ectopically expressed in transgenic cells from MB subgroups, and they were subjected to molecular and phenotypic investigations. Monoclonal cells stably expressing miR-211 were injected into the mouse cerebellum. miR-211 forced expression acts as a tumor suppressor in MB both in vitro and in vivo, attenuating growth, promoting apoptosis, and inhibiting invasion. In support of emerging regulatory roles of metabolism in various forms of cancer, we identified the acyl-CoA synthetase long-chain family member (*ACSL4*) as a direct miR-211 target. Furthermore, lipid nanoparticle-coated, dendrimer-coated, and cerium oxide-coated miR-211 nanoparticles were applied to deliver synthetic miR-211 into MB cell lines and cellular responses were assayed. Synthesizing nanoparticle-miR-211 conjugates can suppress MB cell viability and invasion in vitro. Our findings reveal miR-211 as a tumor suppressor and a potential therapeutic agent in MB. This proof-of-concept paves the way for further pre-clinical and clinical development.

**Graphical Abstract:**

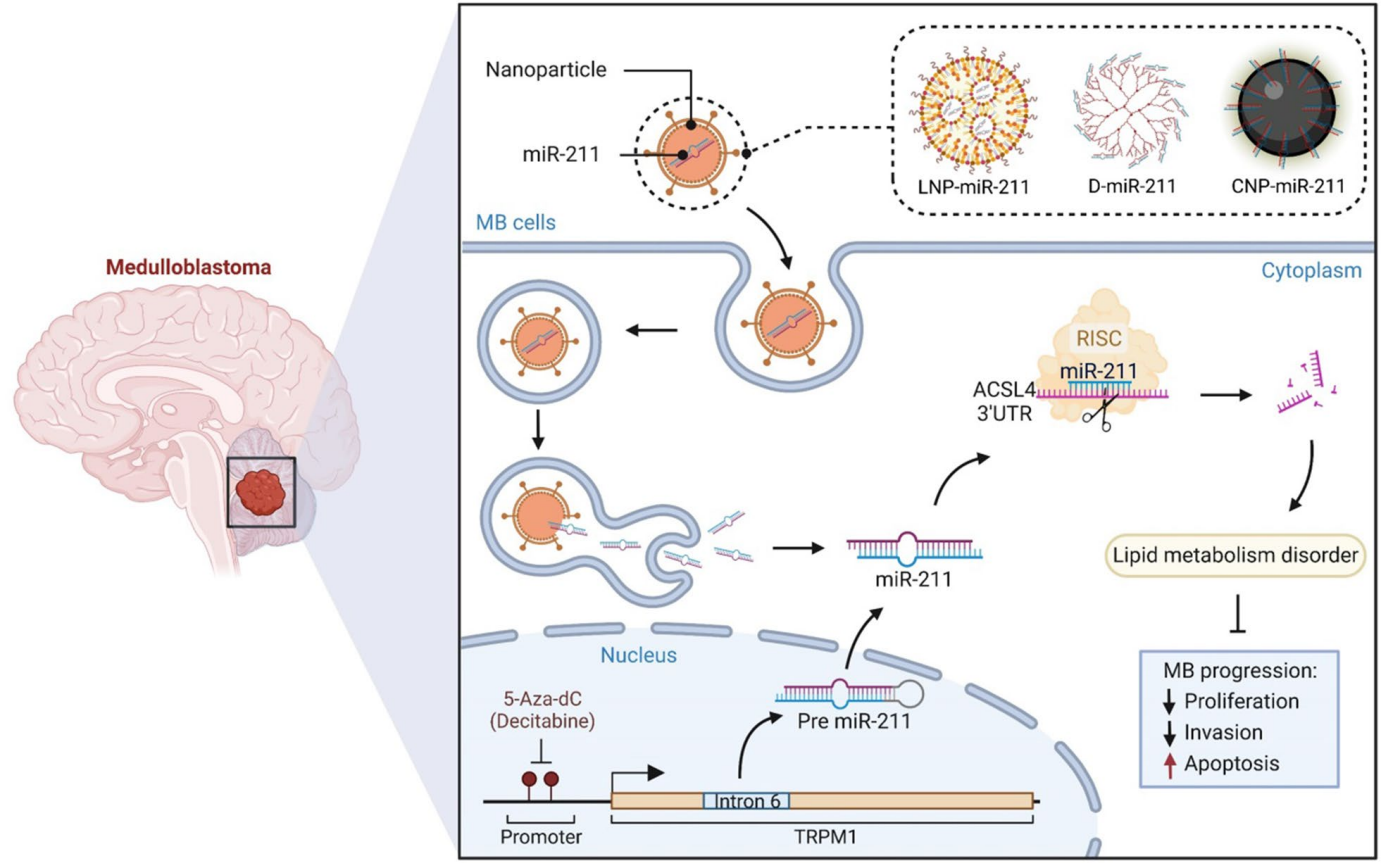

**Supplementary Information:**

The online version contains supplementary material available at 10.1186/s40478-023-01684-w.

## Introduction

Brain tumors are the second most common malignancy in childhood and the leading cause of cancer‐related morbidity and mortality in the pediatric age group. Medulloblastoma (MB) accounts for 20% of pediatric intracranial embryonal tumors [33, 36], and although advances in surgery and adjuvant therapies have increased long-term survival, approximately 30% of MB patients eventually develop recurrences and metastases [14, 51]. Furthermore, the highly toxic side effects of radiation and chemotherapy often leave surviving children with a poor quality of life. There is an urgent need for new, safer therapies based on a thorough understanding of the cellular and molecular architecture of MBs. Furthering this aim, molecular analyses have subdivided MB into at least four principal molecular groups—WNT‐activated, SHH-activated, Group 3, and Group 4 [28, 34]—which differ in their underlying genetic alterations, clinical features, and treatment, with patients categorized into non-WNT subgroups having worse overall survival rates [28, 35, 41].

The human genome includes an abundance of non-protein-coding transcripts that are as central as proteins for regulating cellular function and identity. miRNAs are small (18–24 nucleotide) non-coding (nc)RNAs that post-transcriptionally regulate the expression of several mRNA targets. Dysregulated miRNA expression crucially impacts fundamental biological processes including cell growth, motility, apoptosis, metabolism, and homeostasis, contributing to cancer development [10, 26]. They are also dysregulated in MB through various mutations, deletions, or amplifications [24, 41, 52]. Large-scale expression profiling and deep-sequencing approaches have revealed that miRNAs play pivotal roles in MB progression, but their full repertoire and impact in MB are unknown [9, 21].

miR-211 is an miRNA implicated in cancer, with pleiotropic effects governing both tumor progression and repression in melanoma [23], colorectal cancer [7], breast cancer [8], and glioma [56]. It is also a brain-enriched miRNA that plays a crucial role in neuronal differentiation and activity [2, 12], allowing us to hypothesize that it might also play a role in MB development. Indeed, miR-204, a highly homologous miRNA to miR-211 with the same seed sequence as the mature species, has been established as a valuable risk stratification marker for group G3 and G4 MBs [4]. miR-204 and miR-211 are encoded within the introns of genes at different chromosomal loci: miR-204 on 9q21.12 and miR-211 on 15q13.3, and their respective host genes, *TRPM3* (miR-204) and *TRPM1* (miR-211), are both important for cellular identity and morphogenesis of neural crest cell-derived cells. This intriguing encoding might explain the distinct but high structural similarity of the two miRNAs, and the phenotypic consequences of miR-211 and miR-204 may also overlap. However, the molecular mechanisms and phenotypes of miR-211 in MB are currently unknown.

We therefore we explored miR-211’s expression and signaling in MB. In doing so, we establish that miR-211 is downregulated in experimental and clinical samples of MB compared with normal cerebellum. miR-211 has a tumor suppressor function, attenuating growth, promoting apoptosis, and inhibiting migration of MBs in vitro and in vivo*.* We identify acyl-CoA synthetase long-chain family member (*ACSL4*) as an miR-211 target, explaining the observed downstream effects of miR-211 on lipid metabolism through a tumor suppressive lipid phenotype of reduced long-chain fatty acids and increased polyunsaturated fatty acids. The metabolic consequences of miR-211 expression include wider effects on cellular metabolism, preserving essential amino acid usage and mitochondrial numbers to maintain oxidative phosphorylation and sensitivity to external cellular stressors. Finally, we demonstrate that miR-211 has potential as a therapeutic for the treatment of MB by synthesizing and delivering nanoparticle-miRNA conjugates into human MB cells in culture, which suppressed viability and cell migration.

## Materials and methods

### RNA samples

RNA was isolated from normal human cerebellum cells (BioChain, Newark, CA), MB cell lines, and patient-derived xenografts (PDXs). The DAOY, ONS-76, D341, D458, D283, and HDMB03 cell lines were kind donations from the Wechsler-Reya and Raabe labs, while DMB006, DMB012, RCMB28, RCMB32, RCMB38, RCMB40, RCMB45, and RCMB51 PDXs were from the Wechsler-Reya lab and MED511FH and MED1712FH from the Olson lab (Fred Hutchinson Cancer Research Center). The Institutional Review Board (IRB) at each institution approved the protocol for PDX collection, and all patients provided written informed consent.

### Cell lines and cell culture

Human MB cell lines CHLA01 and DAOY were purchased from the American Type Culture Collection, and D425 was purchased from Sigma-Aldrich (St. Louis, MO). STR profiling and *Mycoplasma* testing were performed on all cell lines. DAOY cells were cultured in Dulbecco’s Modified Eagle medium (Gibco, Thermo Fisher Scientific, Waltham, MA) supplemented with 10% fetal bovine serum (FBS, Gibco), 1% sodium pyruvate, non-essential amino acids, and penicillin/streptomycin. D425 cells were cultured in DMEM/F12 with 10% FBS and 1% penicillin/streptomycin. CHLA01 cells were cultured in DMEM/F12 with B-27 supplement (Invitrogen, Waltham, MA), 20 ng/ml bFGF (R&D Systems, Minneapolis, MI), and 20 ng/ml EGF (R&D Systems). Cells were grown in a humidified incubator at 37 °C in 5% CO_2_ and culture medium was replaced every 3 to 4 days. The cells were gently trypsinized (0.05%, Gibco) for subculture.

### Quantitative real-time PCR

As per the manufacturer's instructions, total RNA was purified using the Direct-zol RNA Miniprep kit (Zymo Research, Irvine, CA). RNA yields were measured with a NanoDrop 8000 spectrophotometer (Thermo Fisher Scientific). For miRNA, we reverse transcribed 10 ng RNA to cDNA. qRT-PCR analysis was performed using TaqMan Universal Master Mix assays (Applied Biosystems, Waltham, MA) using TaqMan primer probes for miR-211 quantification and RNU48 primer probe as control. For mRNA, we reverse transcribed 500 ng RNA using High-Capacity cDNA Reverse Transcription Kits (Applied Biosystems) before performing quantitative PCR using SYBR Green Master Mix assays (Applied Biosystems). The primer sequences are listed in Additional file 3: Table 1. For genomic DNA, total DNA was isolated using the Quick DNA Miniprep kit (Zymo Research) as per the manufacturer’s protocol. qRT-PCR analysis was performed using 100 ng mitochondrial and genomic DNA.

### Bulk RNA sequencing

Libraries were constructed using the TruSeq Stranded Total RNA Library Prep Gold kit following the manufacturer’s protocol (Illumina) from the total RNA extracted from MB cells. The quality of DNA libraries was evaluated using the KAPA library qualification kit (Roche) and Agilent 2100 Bioanalyzer (Agilent Technologies). Libraries were normalized to 2 nM and pooled libraries subjected to 75-nucleotide deep sequencing using the Illumina NextSeq 550 system to obtain a minimum of 36 million reads per library. Reads were aligned to the reference human genome hg38 using STAR aligner. Expression quantification was performed using RSEM algorithm. Data were normalized by variance-stabilizing transformation using DESeq2 software, which considers the RNA-seq data size of each sample.

### Western blotting

Cells were lysed on ice using RIPA buffer (Thermo Fisher Scientific) with a cocktail of proteinase inhibitors. Protein lysates were electrophoresed on 10% SDS polyacrylamide gels before transfer to PVDF membranes (MilliporeSigma, Burlington, MA). Membranes were blocked with 5% skimmed milk and incubated with primary antibodies: anti-*ACSL4* (Abcam, ab155282), anti-*RAB22A* (Proteintech, 12125-1-AP), anti-*SERINC3* (LSBio, LS-C386356), or anti-*GAPDH* (GeneTex, GTX100118) overnight at 4 °C. Subsequently, goat anti-rabbit HRP antibodies (1:5000, Bio-Rad) were applied for 1 h at room temperature. After washing with 1% TBST, membranes were developed with enhanced chemiluminescence (SuperSignal West Pico PLUS) solution (Thermo Fisher Scientific) and exposed to a ChemiDoc imaging system (Bio-Rad, Hercules, CA). Data were analyzed using ImageJ software (NIH).

### Cell proliferation assay

Cells were harvested in the logarithmic phase and trypsinized with 0.05% trypsin. For the cell proliferation assay, cells were cultured in 96-well plates for varying times. 20 µl MTS solution (Promega) was added to each well and, 2 h later, the absorbance (optical density value) was measured at a 490 nm on the EnVision 2105 microplate reader (PerkinElmer).

### Clonogenic and soft agar colony formation assays

For the clonogenic assay, we seeded DAOY cells in six-well plates at a density of 2 × 10^3^ cells/well for approximately 14 days. Then, cells were fixed with 4% paraformaldehyde and stained with 0.5% crystal violet solution. For the soft agar colony formation assay, we suspended D425 and CHLA01 cells in growth medium containing 0.6% agar and plated 1 × 10^3^ cells in 24-well plates (1 ml/well) on top of a layer of growth medium containing 1.2% agar (1.5 ml/well). Agar and growth medium with FBS (20%) were added. Cells were incubated for two weeks at 37 °C, and the formation of viable colonies quantified using the Cytation 1 cell imaging reader (BioTek, Winooski, VT). Colony number was determined using Image Pro Plus (Media Cybernetics, Rockville, MD).

### Apoptosis assay

Following the manufacturer's protocol, apoptotic cells were analyzed using a fluorescein isothiocyanate (FITC) Annexin V Apoptosis Detection Kit (BD Biosciences). Cells were collected, washed, and stained with FITC-annexin V and propidium iodide (PI) and apoptosis detected by CytoFLEX LX flow cytometer (Beckman Coulter, Brea, CA). Data were quantified using CytExpert 2.4 software.

### Invasion assay

For transwell invasion assays, Matrigel invasion chambers for 24-well plates (membrane 8.0 μm pores) were purchased from Corning (Corning, NY). Cells were resuspended in 100 μl serum-free medium and plated in the upper chamber with D425 and CHLA01 1 × 10^5^ cells/well or DAOY 0.2 × 10^5^ cells/well. 600 μl complete medium was added into the lower chamber. After 48 h at 37 °C, paraformaldehyde was used to fix the cells invading the lower chamber before washing in PBS and staining with 0.5% crystal violet solution. Cell numbers were counted using ImageJ software (NIH).

### Targeted metabolomics

MB cells, including 10^6^ D425 with vector-only or ectopic miR-211 cells, were subjected to targeted metabolomics. D425 cells were centrifuged with vector-only or ectopic miR-211 cells and flash-frozen in liquid nitrogen. Cell pellets were stored on dry ice and delivered to the metabolomics core facility at Sanford Burnham Prebys Medical Discovery Institute (SBP). Samples were thawed on ice, vortexed, protein concentrations determined, and prepared for analysis according to the core’s solvent extraction methods. Metabolomics were assessed using liquid chromatography/mass spectroscopy (LC/MS), and metabolites were identified by accurate mass searching and MS/MS spectral matching with an in-house standard MS/MS library. We analyzed all cell samples in triplicate.

### Nanoparticles and miR-211 synthesis

Double-stranded miR-211 was synthesized by Synbio Technologies. An ionizable lipid nanoparticle (LNP) was prepared using microfluidic mixing. Dendrimer-miR-211 conjugates were synthesized using a multistep reaction protocol. The PAMAM-G6-OH (D6-OH) dendrimer composed of ~ 256 terminal hydroxyl groups were used for this synthesis. The cerium oxide nanoparticle (CNP) was synthesized using wet-chemical hydrolysis method at room temperature. Details can be found in the Additional file 2.

### Cellular uptake of nanoparticle-miR-211

We seeded D425 cells in six-well plates at a density of 5 × 10^5^ cells per well and then added fresh medium containing LNP-, dendrimer-, or dendrimer-miR-211 complexes at different concentrations in each well. After 12–96 h incubation, cells were collected and washed twice with PBS. The cellular uptake of nanoparticle-miR-211 complexes was acquired with an CytoFLEX LX flow cytometer (Beckman Coulter). The Cy3 fluorescence intensity of cells was detected, and data were quantified using CytExpert 2.4 software. Cells treated for 12–96 h were harvested for RNA extraction.

### Nanoparticle-miR-211 treatment

We prepared D425 cells as described above and treated them with LNP-, dendrimer-, or CNP-miR-211 complexes, followed by cell proliferation, apoptosis, and invasion assays. Cells treated with LNP-, dendrimer-, or CNP under the same processes were used as control.

### Animal experiments

All animal studies were approved and performed in accordance with the policies and regulations of the Animal Care and Use Committee of Johns Hopkins University. NOD-SCID nude mice were obtained from the Jackson Laboratory (Bar Harbor, ME), 4–6 weeks old, and maintained under specific pathogen-free (SPF) conditions in the animal care facility. We infected cells engineered to stably express empty vector or miR-211 with lentiviruses carrying renilla luciferase construct. Cerebellar coordinates were -2 mm from lambda, + 1 mm laterally, and 1.5 mm deep. Tumor growth was evaluated by weekly intraperitoneal injection of Viviren (50 μg/mouse in PBS, Promega). After injection at 10–15 min, bioluminescence was captured with an in vivo spectral imaging system (IVIS Lumina II, Xenogen, Alameda, CA). We collected tumor tissue after euthanizing mice on days 35–42.

### Statistical analysis

All data are presented as mean ± SD. GraphPad Prism and SPSS 17.0 were used for statistical analysis. Student’s *t*-test was used to analyze differences between two groups, while the Kruskal–Wallis test was used to evaluate differences between more than two groups. A *P*-value < 0.05 indicated statistical significance.

### Data availability

RNA-seq data described in the manuscript is accessible at NCBI GEO accession number GSE188813 and GSE240544.

## Results and discussion

### miR-211 is downregulated in medulloblastoma

**Fig. 1:**
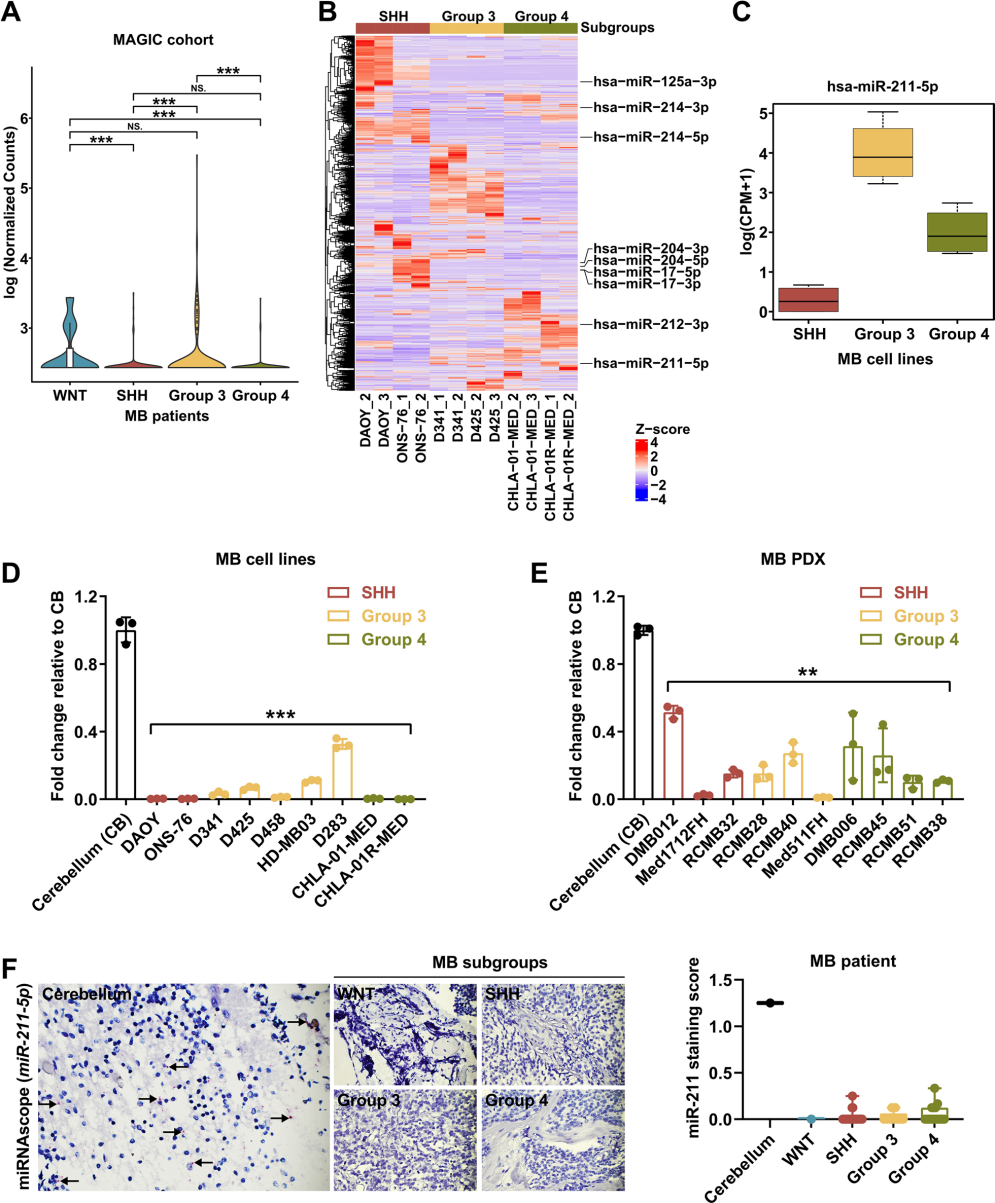
miR-211 expression is downregulated in MB. **A** Violin plot showing distribution of normalized expression of miR-211 in the 4 molecular subgroups (WNT: 11, SHH: 250, Group 3: 219, Group 4: 326) of MB patients from the MAGIC cohort (n = 806). **B** Heatmap of differential miRNA expression in six MB cell lines (SHH: DAOY and ONS-76, Group 3: D341 and D425, Group 4: CHLA-01-MED and CHLA-01R-MED). Gene expression data were obtained using human small RNA-seq. Expression values shown are z-scores. **C** Normalized expression of miR-211 in the MB cell lines from small RNA-seq. **D**, **E** Expression of miR-211 in SSH, Group 3, and Group 4 MB cell lines (D) and patient-derived xenograft (PDX) samples **E** was detected by qRT-PCR. Values indicate fold change relative to normal cerebellum (CB). **F** Representative images of CISH for miR-211-positive normal cerebellum tissue (left panel). Scatter plot showing miR-211 staining score in normal cerebellum and different MB subgroups (right panel). Magnification × 400. Arrowhead, miR-211-positive cells. Data, mean ± SD. **P < 0.01, ***P < 0.001

To investigate the role of miR-211 in human MB, we first examined its expression in the MAGIC cohort of 806 bulk RNA-seq samples (WNT: 11, SHH: 250, Group 3: 219, Group 4: 326) and revealed low miR-211 expression mainly in SHH and Group 4 MB subgroups (Fig. 1A). We also examined another closely related miRNA, miR-204, (Additional file 1: Fig. 1A), which although nearly identical to miR-211, shows lower expression only in SHH MB subtypes (Additional file 1: Fig. 1B and C). Next, we performed small RNA sequencing in SHH, G3, and G4 MB cell lines (SHH: DAOY and ONS-76; Group 3: D341 and D425; Group 4: CHLA-01-MED and CHLA-01R-MED) (Fig. 1B and C), which showed distinct small RNA expression profiles in the different molecular subgroups and low miR-211 expression in SHH samples, medium expression in G4 samples, and high expression in G3 samples, mirroring the expression patterns seen in clinical samples. By qRT-PCR of cell line (Fig. 1D) and patient-derived xenograft (PDXs) samples (Fig. 1E), miR-211 levels were consistently and significantly lower than in normal cerebellum samples. To confirm miRNA expression in clinical tissue samples, we developed an RNA-CISH assay to detect miR-211 in patient tissues (n = 55; WNT: 1, SHH: 29, Group 3: 9, Group 4: 16) and normal cerebellum (n = 1) (Fig. 1F), which confirmed that miR-211 was expressed in scattered cells of the normal cerebellum but not in MB samples, again mirroring the expression seen in cell lines, PDXs, and clinical samples. Interestingly, recent single-cell sequencing studies [18] have demonstrated different cells of origin of different MB subgroups, which might explain the different levels of miR-211 seen in different subgroups.

miR-211 is an intronic RNA in the sixth intron of transient receptor potential cation channel subfamily M member 1 (*TRPM1*), or melastatin [32]. The encoded protein is a calcium-permeable cation channel expressed in melanocytes. We were the first group to report the importance of miR-211 in melanocytes and in melanomas [1, 23, 30, 31, 37, 40, 42, 43], demonstrating that the *TRPM1* promoter regulates miR-211. To evaluate whether the *TRPM1* promoter regulates miR-211 in MB, we applied MethPrimer, a CpG island prediction program, and identified CpG-rich regions (− 1247 to − 1042 bp) in the upstream region of the transcriptional start site of the *TRPM1* promoter (Additional file 1: Fig. 2A). Treating D425 MB cells with the DNMT inhibitor 5-Aza-dC (IC_50_ 14.8 μM; Additional file 1: Fig. 2B), we detected dose-dependent induction of *TRPM1* mRNA and miR-211 expression (Additional file 1: Fig. 2C). Interestingly, D425 cells overexpressing miR-211 treated with 5-Aza-dC showed the greatest inhibition of proliferation (Additional file 1: Fig. 2D).

Since the *TRPM1* gene regulates miR-211, we decided to examine *TRPM1* expression in ICGC and PDX samples, predicting that its expression would be lower in cancer samples than in normal cerebellum. As expected, *TRPM1* expression was lower in ICGC and PDX samples than in the cerebellum (Additional file 1: Fig. 3A and B), consistent with the *TRPM1* promoter regulating miR-211 expression in the MB context.

### miR-211 acts as a tumor suppressor in MB both in vitro and in vivo

**Fig. 2:**
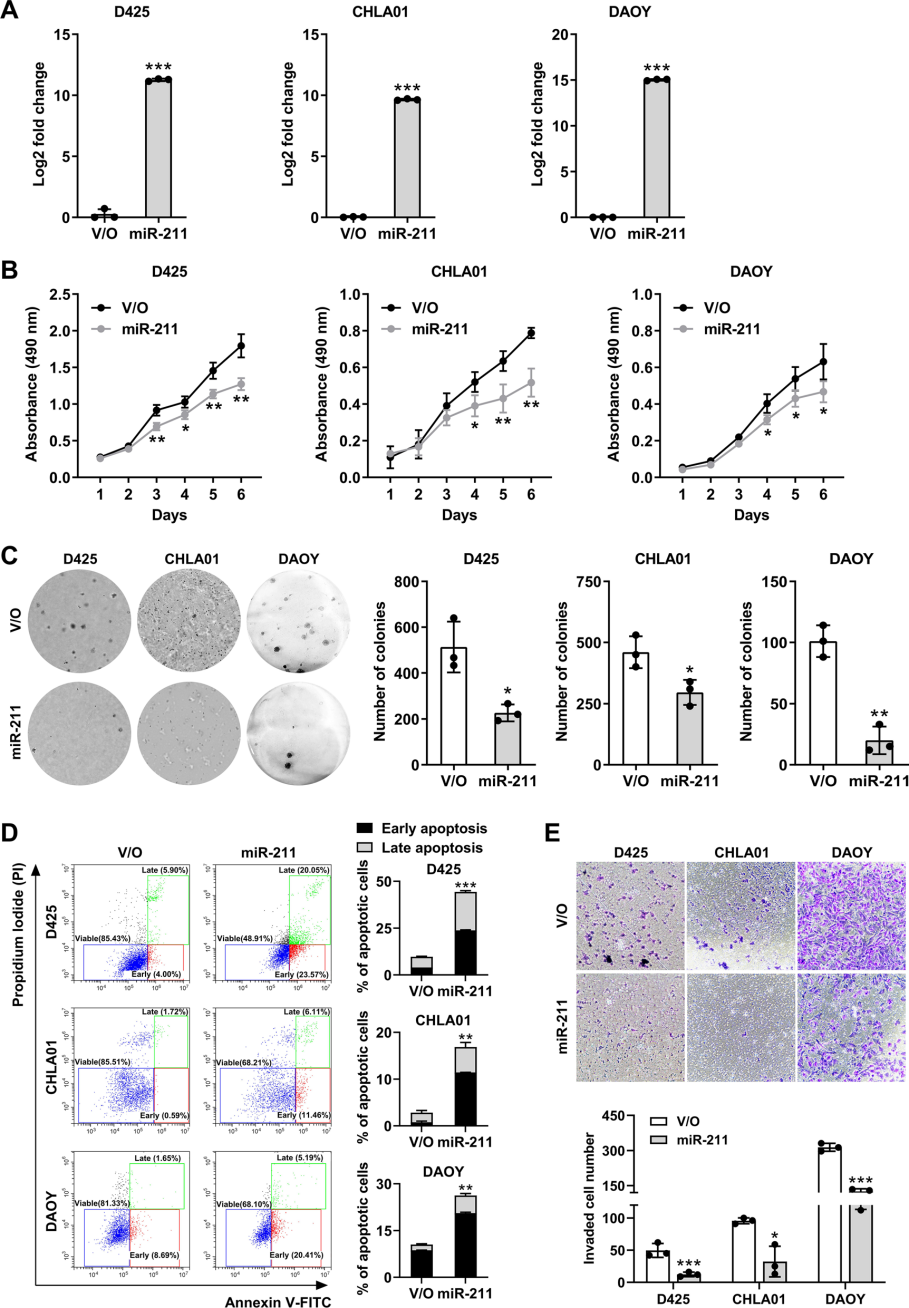
Effects of miR-211 expression on the proliferation, apoptosis, and invasion of MB cells. **A** Expression of miR-211 in MB cells expressing vector only (V/O) or miR-211 was detected by qRT-PCR. **B** The effect of miR-211 overexpression on the viability of human MB cells by the MTS assay. **C** Stable MB cells expressing vector only or miR-211 were subjected to soft agar and colony formation analysis. **D** Apoptosis was determined by Annexin V/PI assays in MB cells expressing vector only (V/O) or miR-211. **E** The effect of miR-211 overexpression on the invasive potential of human MBs cells in transwell assays. Data, mean ± SD. *P < 0.05, **P < 0.01, ***P < 0.001

To test the hypothesis that miR-211 acts as a tumor suppressor in MB as in some other cancers [25, 38, 39], we first introduced synthetic miR-211 under a constitutive promoter into three MB cell lines representing SHH, G3, and G4 MBs (DAOY, D425, and CHLA-01-MED, respectively), as confirmed by qRT-PCR analysis (Fig. 2A). Cell viability assays revealed that miR-211 suppressed the viability (Fig. 2B) and colony formation (Fig. 2C) of all three MB cell lines. Flow cytometry analysis revealed increased apoptosis of D425, CHLA01, and DAOY cells after miR-211 overexpression (Fig. 2D), with the highest early apoptosis (> 20%) seen in G3 MB (D425) and SHH (DAOY) cell lines. In a transwell invasion assay, miR-211 overexpression suppressed MB cell invasion (Fig. 2E), especially in SHH subgroup cells. Therefore, miR-211 may act as a tumor suppressor in MB.

**Fig. 3:**
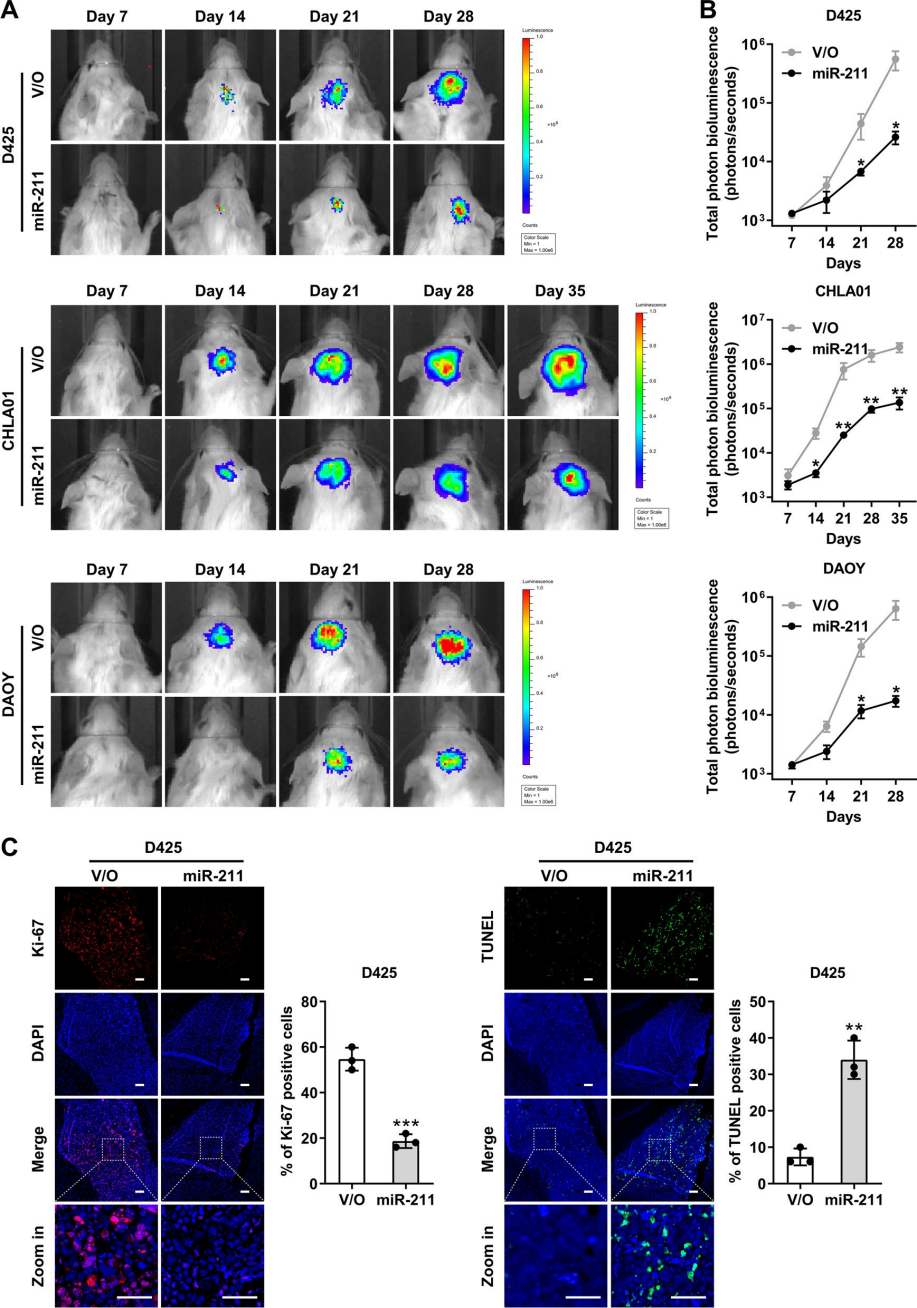
miR-211 inhibits the tumorigenicity of MB cells. **A** MB cells expressing vector only (V/O) or miR-211 were stably transfected with a renilla luciferase expressing construct and implanted into the cerebella of NOD-SCID mice. Tumor formation was assessed weekly after tumor implantation by bioluminescent signal detection with the IVIS platform. **B** Quantification of total photon counts from mice implanted with MB cells expressing V/O or miR-211. **C** Fluorescent staining of Ki-67 and TUNEL in the D425 xenografts on day 35 after intracranial injection. Images in the lower column are the magnified images within the white boxes found in the middle column. Nuclei are stained with DAPI (blue). Scale bars, 100 μm. Data, mean ± SD. *P < 0.05, **P < 0.01, ***P < 0.001

To test whether miR-211 suppresses tumorigenicity in mice in vivo*,* we transplanted D425, CHLA01, and DAOY parental cells and the corresponding miR-211-overexpressing cells intracranially to establish cerebellar xenografts. In vivo imaging (IVIS platform) showed that cells overexpressing miR-211 produced significantly smaller tumors than their parental counterparts (Fig. 3A), decreasing tumor volumes by 30-fold (Fig. 3B). Furthermore, higher miR-211 expression was associated with reduced in vivo proliferation according to Ki67 expression and increased apoptosis by TUNEL (Fig. 3C, Additional file 1: Fig. 4A and B), suggesting that miR-211 acts as a potent tumor suppressor in MB.

### *ACSL4* is a miR-211 target gene that reprograms lipid metabolism

**Fig. 4:**
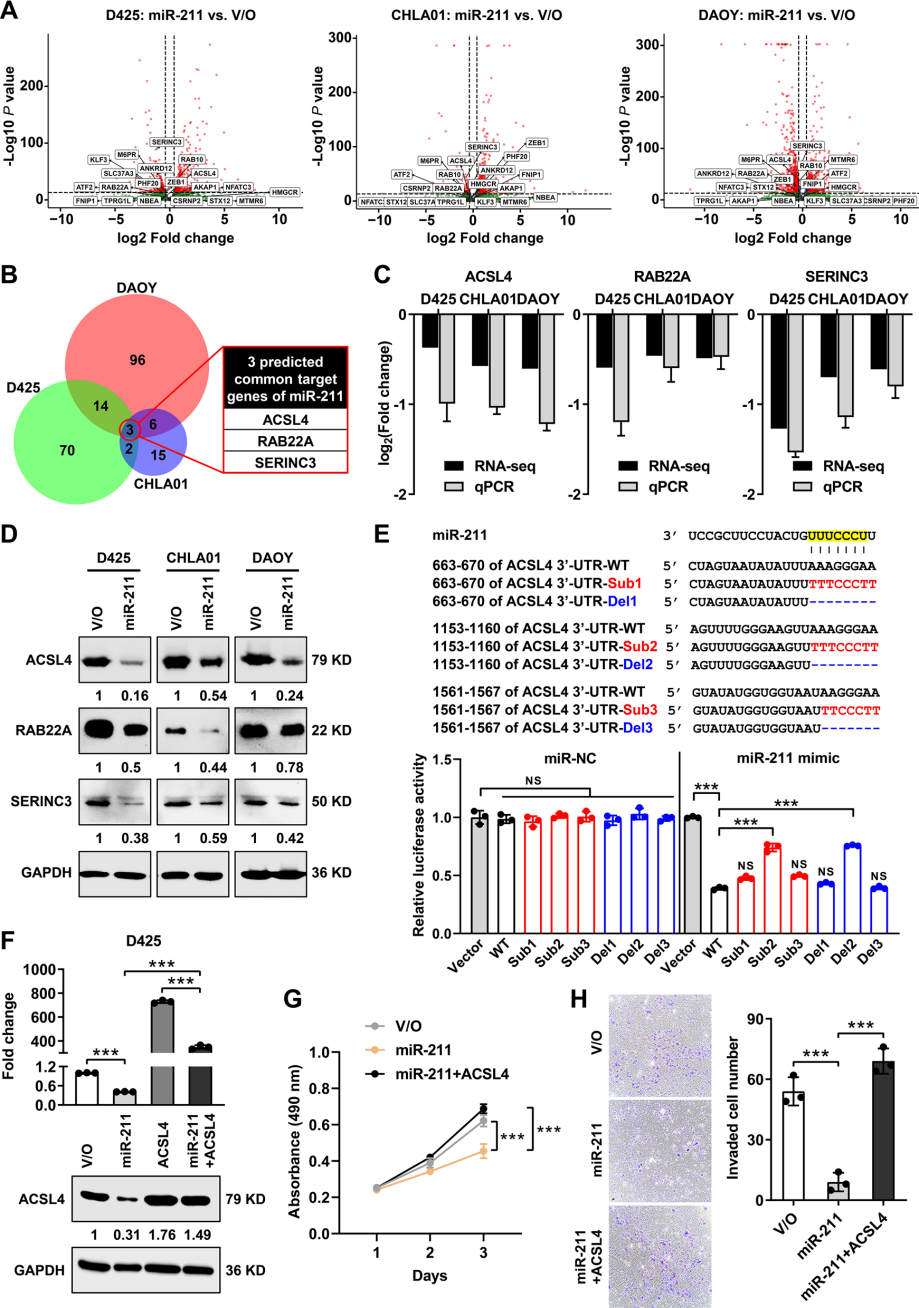
miR-211 inhibits MB progression by targeting *ACSL4* expression. **A** Volcano plot of differentially expressed genes in MB expressing miR-211 or vector only (V/O) cells. **B** Venn diagram revealing the overlap of potential target genes based on miR-211 targets identified in the TargetScan database and the RNA-seq data of downregulated genes from MB cells. **C** Expression levels of potential miR-211 targets *ACSL4*, *RAB22A*, and *SERINC3* in MB cells expressing vector only (V/O) or miR-211 were validated by qRT-PCR. **D** Relative protein levels of *ACSL4*, *RAB22A*, and *SERINC3* were detected by western blotting after miR-211 overexpression in MB cells. **E** Luciferase activity assay for targeting sequences of the wild or mutant *ACSL4* 3′-UTR by miR-211 in D425 cells. **F** D425 cells expressing miR-211 or vector only (V/O) were transiently transfected with *ACSL4* plasmid, and lysates were subjected to qRT-PCR and western blotting. **G** Cell viability was determined by MTS assays in D425 cells overexpressing miR-211 with and without introduction of *ACSL4* plasmid. **H** Cell invasive potential was determined by transwell assays in D425 cells overexpressing miR-211 with and without introduction of *ACSL4* plasmid. Data, mean ± SD. ***P < 0.001, NS, non-significant

To identify miR-211 target genes that might inhibit tumor growth in vitro and in vivo, we sequenced parental MB cells and corresponding miR-211-overexpressing cells. miR-211 overexpression downregulated 1,736 transcripts in D425 cells, 447 transcripts in CHLA01 cells, and 2,346 transcripts DAOY cells relative to their respective vector-only parental cells (Fig. 4A, Additional file 4: Table 2). By predicting miR-211 targets in genes downregulated in all three cell lines, we identified three candidate miR-211 targets (Fig. 4B): acyl-CoA synthetase long-chain family member 4 (*ACSL4*), Ras-related protein (*RAB22A*), and serine incorporator 3 (*SERINC3*). The mRNA and protein expression levels of *ACSL4*, *RAB22A*, and *SERINC3* are illustrated in Fig. 4C and D.

Of these putative miR-211 targets, the long-chain acyl-CoA synthetases (ACSLs) regulate the balance between anabolic and catabolic pathways. Given this potentially global effect on cellular function and that miR-211 has previously been implicated in metabolic regulation [31, 43, 45, 55], we chose to investigate *ACSL4* further. Acyl-CoA synthetase long-chain family member 4 (*ACSL4*) encodes one of five ACSL isoforms and is unique in that it is present primarily in peroxisomes and mitochondria-associated membranes [53]. *ACSL4* is dysregulated in many human cancers, with complex pleiotropic roles depending on the tumor type and its microenvironment [17]. For instance, in hepatocellular carcinoma (HCC) [15, 38], *ACSL4* is oncogenic, while in lung and gastric cancer it has a tumor suppressive function [54, 57]. We therefore sought to establish whether *ACSL4* was a direct target of miR-211 in MB, as shown previously in HCC [38].

To identify whether miR-211 and ACSL4 expression level are inversely correlated in MB patients, we interrogated 806 patients RNA-seq data in the MAGIC database. One major challenge in this dataset is that the conventional RNA-seq isn't the best dataset for detecting miRNA expression in MBs. Unfortunately, no dataset has shown a small RNA sequence (their small sizes lead to selective elimination of most miRNA signals relative to the noise in these data sets); miRNA analysis by RNA-seq requires special pre-isolation of the small RNA fractions, which were not done in most of these experiments); therefore, we only had limited datasets to do this analysis. In this limited dataset, WNT and Group 3 MB samples seem to have the highest expression of miR-211: 27% of WNT and 14% of Group 3 MB express miR211. Additional file 1: Fig. 5A depict the expression relationship between miR-211 expression and ACSL4 in all MB (upper panel) and Group 3 MB samples (lower panel). Although the small sample number does not allow a statistically significant correlation, the trend of an expected negative correlation was observed.

Interrogation of the TargetScan database identified *ACSL4* as a high confidence downstream target of miR-211, with the 3′-UTR of *ACSL4* containing three putative miR-211-binding sites (Fig. 4E). We designed six mutations of the *ACSL4* 3′-UTR fused to the 3’ end of a luciferase mRNA and addressed by transfection whether mutations abolished miR-211 targeting activity. miR-211 mimics significantly reduced luciferase activity for *ACSL4* 3′-UTR-WT and increased luciferase activity for *ACSL4* 3′-UTR-Sub2 or Del2, suggesting that miR-211 binds within the *ACSL4* 3′-UTR (Fig. 4E). The other two miR-211 seed sequences (NT 663–670 and NT 1561–1567) did not alter luciferase activity and, therefore, only mutations in the miR-211 recognition site NT 1153–1160 mediated miR-211 binding to the *ACSL4* 3′-UTR. Molecular dynamics (MD) simulation further modeled a complex of human Argonaute2-miR-211 paired with the *ACSL4* target sequence, which revealed stable base pairing between the miR-211 seed and the corresponding 3′-UTR site (NT 1153–1160), as illustrated in Additional file 5.

Next, we hypothesized that *ACSL4* re-expression would overcome miR-211-associated phenotypes. To test the hypothesis, we overexpressed *ACSL4* in MB cells expressing miR-211 (Fig. 4F, Additional file 1: Fig. 5B) and, as expected, found that *ACSL4* reversed miR‐211-driven changes in cell viability and invasion (Fig. 4G and H, Additional file 1: Fig. 5C and D), increasing both to control or even greater levels.

*ACSL4* is a key enzyme in the fatty acid elongation pathway associated with the biosynthesis of long chain fatty acids (LCFAs) or very long chain fatty acid (VLCFAs), dysregulation of which are associated with cancer progression and metastasis. Indeed, ACSL-mediated lipid anabolism may promote cancer growth, since cancer cells require a continuous source of lipids for growth and survival [27, 47]. Since miR-211 overexpression significantly downregulated *ACSL4* expression, we predicted that this loss would regulate downstream lipid metabolism. KEGG pathway analysis of RNA-seq data revealed alterations in several common metabolic processes [20], especially lipid metabolism (Additional file 1: Fig. 6A), as expected. We conducted ultra-high-pressure liquid chromatography coupled with high-resolution mass spectrometry (UHPLC-HRMS) to identify global lipidomic changes in D425, CHLA01, and DAOY cell lines with or without miR-211 overexpression (Additional file 1: Fig. 6B). miR-211 overexpression significantly decreased LCFA and VLCFA fatty acid metabolism in MB cell lines (Additional file 1: Fig. 6C). Furthermore, lipid saturation, including polyunsaturated versus mono or saturated lipids, affects cancer cell growth, with evidence suggesting that polyunsaturated fatty acids (PUFAs) may have cytotoxic or anti-proliferative effects [11]. Consistent with this, miR-211 overexpression also significantly modified lipid saturation in D425, CHLA01, and DAOY cells (Additional file 1: Fig. 6D), activating PUFAs, fatty acids with more than 3 double bonds, and dihydrosphingolipids. In contrast, saturated fatty acids (SFA), monounsaturated fatty acids (MUFA), and fatty acids with less than 2 double bonds were markedly reduced in miR-211 overexpressing cells compared with parental controls (Additional file 1: Fig. 6D). Taken together, these results support that miR-211 targets *ACSL4* to reprogram lipid metabolism to a tumor suppressive lipidomic profile in MB.

### miR-211 is a global metabolic regulator in MB

The reprogramming of energy metabolism is a hallmark of tumor cells during malignant growth [16], with the Warburg effect (increased glucose uptake and preferential lactate production) and glutamine addiction [46, 50] features of a pro-malignant phenotype. Cancer cells must alter major energy supply and consumption pathways to support the increased energy demands of cancer growth and survival. Indeed, analysis of the metabolite profiles of MB tissues and cerebrospinal fluid (CSF) samples from MB patients revealed abnormal levels of several cancer-specific lipids and essential amino acids (EAA) in MB [3, 22]. In addition to lipogenesis, a key aspect of metabolic reprogramming in MB includes a switch to aerobic glycolysis by neural progenitor cells [48], and several studies have shown that disruption of lipogenesis or glycolysis restricts MB growth, perhaps synergistically [13, 49].

**Fig. 5:**
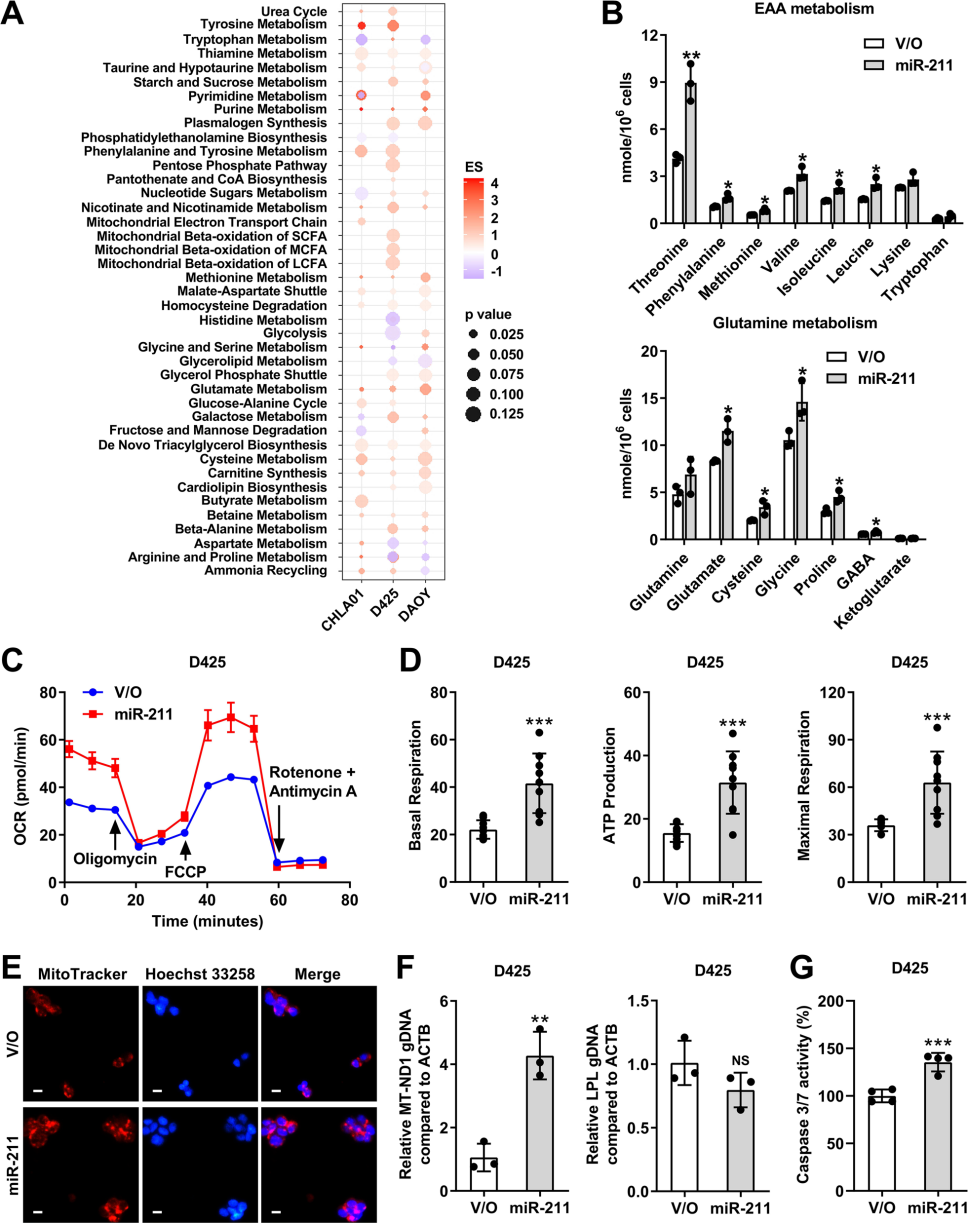
miR-211 is a metabolic regulator in MB cells. **A** Global metabolic network analysis of differentially abundant metabolites in MB expressing miR-211 or vector only (V/O) cells. FDR-corrected *p*-value shown. **B** Quantitative analysis of intermediates for essential amino acid (EAA) and glutamine metabolism in D425 cells expressing miR-211 compared with control. **C** Oxygen consumption rate (OCR) was analyzed using the Seahorse XF analyzer in D425 vector only (V/O) or miR-211 overexpressing cells. **D** Basal OCR were measured at three time points, followed by sequential injections of the ATP synthase inhibitor oligomycin, the uncoupler FCCP, the complex I inhibitor rotenone, and the complex III inhibitor antimycin A. **E** The effect of miR-211 overexpression on the mitochondrial content of human D425 cells by MitoTracker Red staining. Red, MitoTracker; Blue, Hoechst 33,258. Scale bars, 10 μm. (F) Ratio of mitochondrial gene *ND1* and lipoprotein lipase (LPL) to genomic β-actin in D425 vector only (V/O) or miR-211 overexpressing cells. **G** Caspase 3/7 activities were detected in D425 vector only (V/O) or miR-211 overexpressing cells. Data, mean ± SD. *P < 0.05, **P < 0.01, ***P < 0.001, NS, non-significant

To explore the role of miR-211 as a metabolic regulator in MBs, we performed untargeted metabolic profiling in parental and miR-211-overexpressing cells (Additional file 1: Fig. 7A). Global metabolic network analysis identified that miR-211 overexpression enriched several metabolic pathways including those affecting amino acids, oxidation–reduction, energy metabolism, nucleotides, mitochondria-associated networks such as carnitine, and lipid peroxidation (Fig. 5A). Targeted metabolomic analysis identified 41 metabolites, including 20 significantly upregulated metabolites (*P* ≤ 0.05), in D425 cells expressing miR-211 (Additional file 1: Fig. 7B). Using hierarchical enrichment analysis of targeted metabolites to identify dysregulated metabolic pathways, we found that miR-211 preferentially affected pathways associated with amino acid metabolism, including pathways for phenylalanine, tyrosine, and tryptophan biosynthesis and for alanine, aspartate, and glutamate metabolism (Additional file 1: Fig. 7C). Essential amino acids (EAAs) not only provide fundamental building blocks for macromolecular biosynthesis but serve as signaling molecules to induce signaling pathway activation, and tumor cells frequently use EAAs to suppress malignant progression [6, 19]. Compared with controls, D425 cells overexpressing miR-211 exhibited increased demand for six of the eight EAAs (Fig. 5B). Notably, the three EAAs (cysteine, glycine, and glutamate) of glutathione increased significantly, indicating upregulation of glutathione metabolism. Glutamate, proline, and GABA, but not glutamine, were upregulated in D425 cells overexpressing miR-211 (Fig. 5B). Tricarboxylic acid (TCA) and glycolysis intermediates increased, but not significantly (Additional file 1: Fig. 7D).

D425 cells therefore demonstrated a predominantly glycolytic phenotype, while miR-211 overexpression induced an energetic phenotype based on metabolite profiling. Following this, bioenergetics analysis data revealed that miR-211 significantly increased the overall mitochondrial oxygen consumption rate (OCR) and extracellular acidification rate (ECAR) of MB cells (Fig. 5C, Additional file 1: Fig. 7E). Overall, miR-211 overexpression shifted mitochondrial energy derivation toward oxidative phosphorylation (Additional file 1: Fig. 7F), although the ECAR/OCR ratio was slightly lower in miR-211-overexpressing cells (Additional file 1: Fig. 7G). We calculated OCR indexes of basal respiration, ATP production, and maximal respiration, and found a dramatic increase in these indexes in D425 cells overexpressing miR-211 (Fig. 5D). Therefore, miR-211 appears to protect cells from the metabolic adaptations to stress conditions (Warburg effect) that favor cancer progression.

Mitochondria, as the primary source of cellular ATP and other crucial metabolites, could represent a target for miR-211-driven inhibition of MB progression via effects on cell bioenergetics [44]. The mitochondria of MB cells overexpressing miR-211 were increased in number (Fig. 5E). There was a significant increase in mitochondrion-specific gene *ND1*, while nuclear LPL DNA levels were the same, indicating miR-211 increased the number of mitochondrial genomes (Fig. 5F). Mitochondria play a vital role in apoptosis, and a greater mitochondrial content impacts apoptotic protein expression to cause cell death [29]. We found that miR-211 overexpression induced caspase 3/7 protein activity in D425 cells (Fig. 5G), suggesting that the increased mitochondrial numbers in D425 cells overexpressing miR-211 may lower the apoptotic threshold, again contributing to tumor suppression.

### Nanoparticle-coated miR-211 has anti-tumor effects in MB

**Fig. 6:**
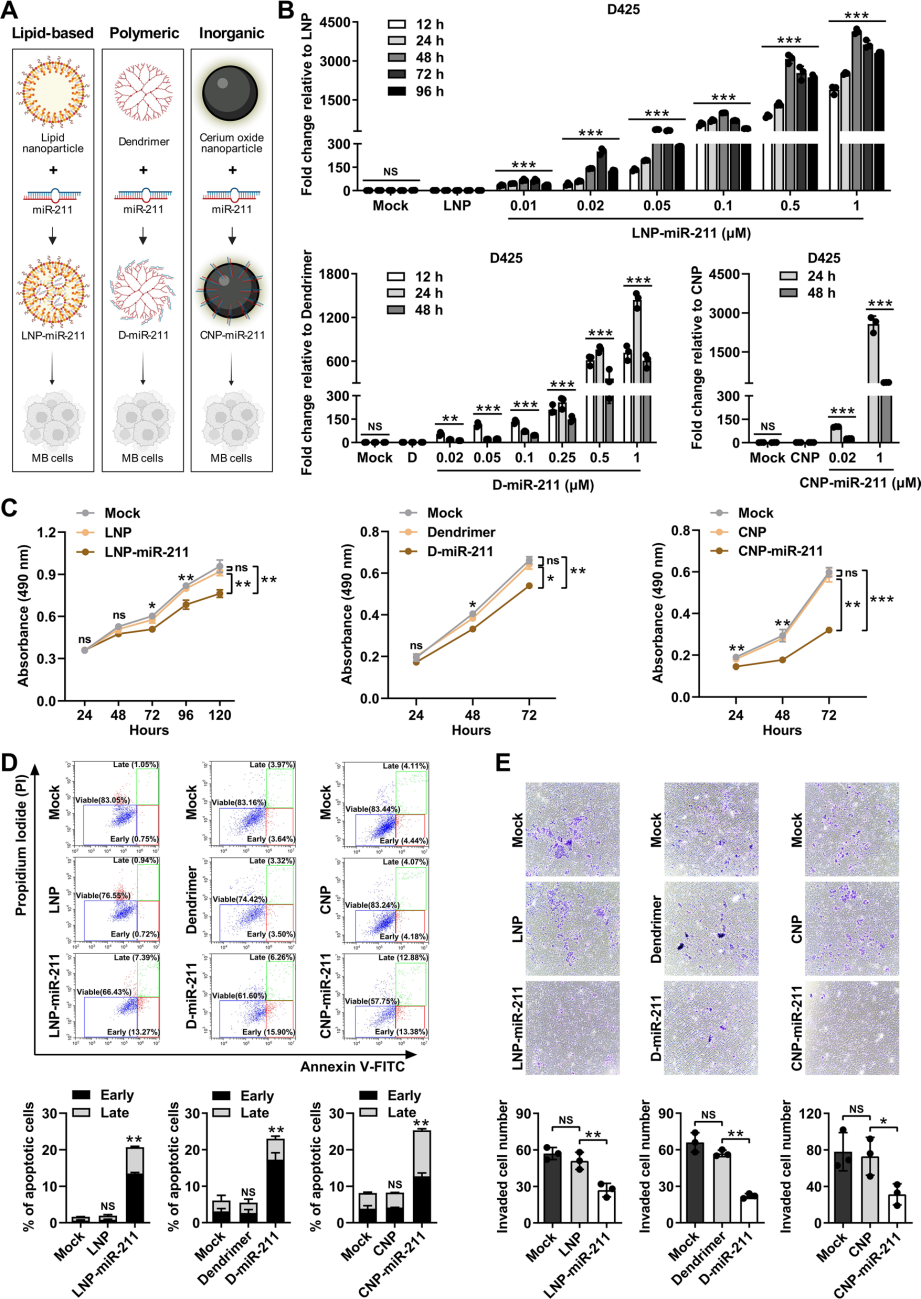
Synthesis and anti-tumor effect of nanoparticle-miR-211 conjugates in MB cells. **A** Schematic of lipid-based (lipid), polymeric (dendrimer), or inorganic (cerium oxide)-miR-211 conjugate synthesis. **B** Expression of miR-211 in D425 cells treated with nanoparticle-miR-211 conjugates at different concentrations and time points by qRT-PCR. **C** The effect of 1 μM CNP-, dendrimer-, LNP-miR-211 on the viability of D425 cells by MTS assays. **D** Percentages of early and late apoptotic cells were determined by Annexin V/PI assays in D425 cells treated with 1 μM CNP-, dendrimer-, and LNP-miR-211 conjugates. **E** The effect of 1 μM CNP-, dendrimer-, LNP-miR-211 conjugates on the invasion of D425 cells in transwell assays. Data, mean ± SD. *P < 0.05, **P < 0.01, ***P < 0.001, NS, non-significant

The tumor suppressive effects of miR-211 make it an ideal candidate therapeutic. Nevertheless, administering naked miRNAs in vivo is challenging due to a lack of tissue specificity, a short circulatory half-life, and potential off-target effects. However, nanoparticles, by shielding the miRNAs from the microenvironment and protecting against degradation, have been used to conjugate or encapsulate miRNAs, with some success [5]. Therefore, to pave the way for using miR-211 as a therapeutic agent, we synthesized three types of nanoparticles to load miR-211 as cargo: lipid-based (lipid), polymeric (dendrimer), or inorganic (cerium oxide) nanoparticles, and applied them to MB cells (Fig. 6A). Nanoparticles were fluorescent dye-labeled to track their intracellular uptake in D425 cells (Additional file 1: Fig. 8A–E), and D425 cells treated with Cy5-labeled dendrimer nanoparticle-miR-211 showed greater uptake than control in a dose-dependent manner (Additional file 1: Fig. 8F). The similar time and dose-dependent manner was shown on cells transfected DiD-labeled lipid nanoparticle and Cy3-labeled miR-211 (Additional file 1: Fig. 9). Treatment with as little as 0.02 μM miR-211-carrying nanoparticles increased miR-211 intracellular level, with maximal expression observed at 1 μM (at least 1400-fold over nanoparticles alone). miR-211 levels peaked at 24 h (CNP- and dendrimer-miR-211) or 48 h (LNP-miR-211) and then gradually decreased in a time-dependent manner. Nevertheless, levels remained higher at 48 h (CNP- and dendrimer-miR-211) or 96 h (LNP-miR-211) in treated cells than in nanoparticle alone control cells (Fig. 6B). Although intracellular miR-211 levels were increased after treatment with 0.02 μM nanoparticle-miR-211 conjugates, a potent inhibitory effect on cell viability was not observed until concentrations reached 1 μM (Fig. 6C, Additional file 1: Fig. 10A–C). Functional studies also revealed an increase in apoptotic cells and a decrease in invasive cells in D425 cells after 1 μM nanoparticle-miR-211 conjugate treatment (Fig. 6D and E). These results suggest that treatment with nanoparticle-miR-211 conjugates leads to a robust anti-tumor effect in MB cells. In vivo nanoparticle delivery and efficacy studies are ongoing in our laboratory.

## Conclusions

Despite their established central role in cancer pathophysiology, the full potential of non-coding RNAs has yet to be realized, in part due to a lack of systematic functional characterization in specific tumor types and also due to challenges in therapeutic delivery. This is especially true for patients with MB, who require new and safe therapeutics, especially those patients with the poorer-prognosis subgroups (non-WNT). Here we not only establish that miR-211 is downregulated in non-WNT MB and has a tumor suppressive role but also identify *ACSL4* as a direct target. Through *ACSL4* and probably other as yet unidentified pathways and targets, miR-211 appears to maintain lipidomic and metabolic homeostasis and protect against the cancer cell adaptations that promote proliferation, migration, and resistance to cell death. Our initial efforts in developing miR-211-nanoparticle conjugates sets the scene for further preclinical and clinical testing as a therapeutic.

### Supplementary Information

**Additional file 1**.Supplementary figures and legends.

**Additional file 2**. Supplementary materials and methods.

**Additional file 3**. List of primers for qPCR.

**Additional file 4**. List of target genes of miR-211 in downregulated genes from RNA-seq.

**Additional file 5**. Supplementary Video 1.
