# Supplementary material for: miRNA-211 maintains metabolic homeostasis in medulloblastoma through its target gene long-chain acyl-CoA synthetase 4
Source: Acta Neuropathol Commun. 2023 Dec 19;11:203. doi: 10.1186/s40478-023-01684-w (PMC10729563; doi:10.1186/s40478-023-01684-w)
Supplement: Supplementary file 1 — Additional file 1.Supplementary figures and legends. [file 40478_2023_1684_MOESM1_ESM.docx]

**Supplementary Figures**


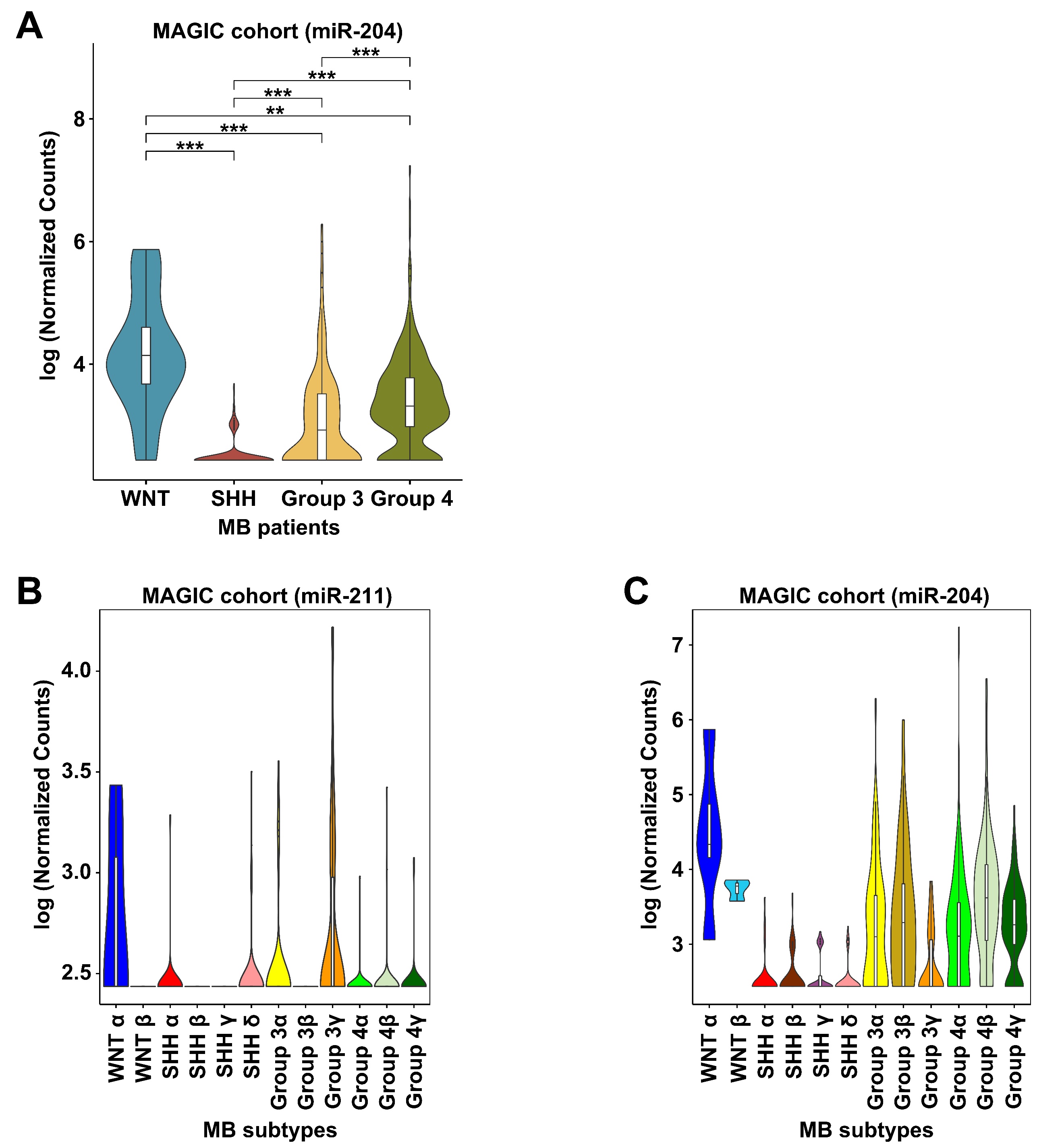


**Supplementary Figure 1. Expression of miR-211 and miR-204 in MB subtypes in the MAGIC cohort.**

(A) Normalized expression of miR-204 in the 4 molecular subgroups (WNT: 11, SHH: 250, Group 3: 219, Group 4: 326) of MB patients classified in the MAGIC cohort (n = 806). (B) Violin plot showing normalized expression of miR-211 in the 12 subtypes of MB patients classified in the MAGIC cohort (n = 806). (C) Violin plot showing normalized expression of miR-204 in the 12 subtypes of MB patients classified in the MAGIC cohort (n = 806). Data, mean ± SD. ***P* < 0.01, ****P* < 0.001,


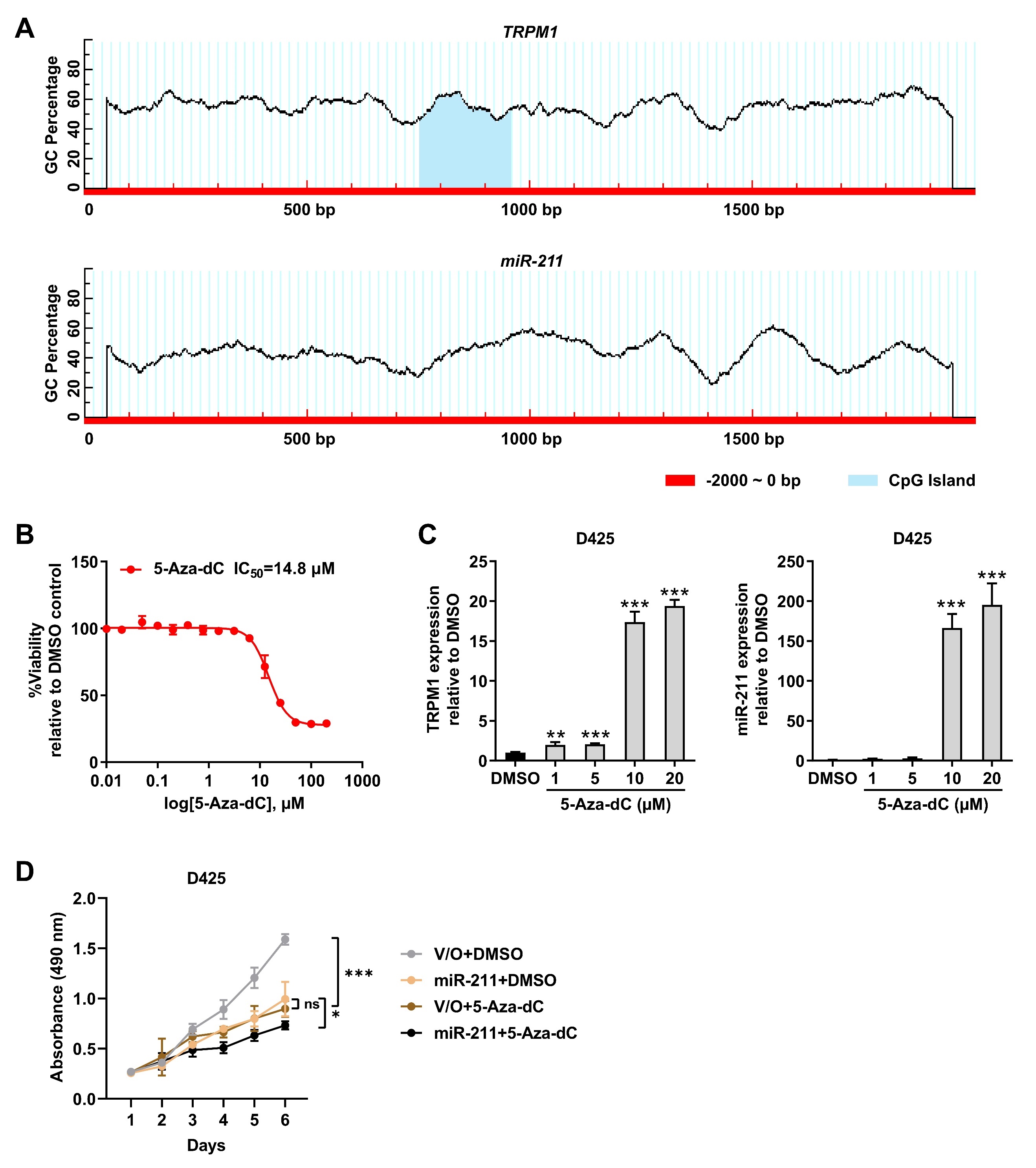


**Supplementary Figure 2. DNA methyltransferase (DNMT) inhibitor increased miR-211 expression and inhibited proliferation in MB cells.**

(A) *TRPM1* and miR-211 promoter sequences were predicted using UCSC. The position of the CpG island was determined using the MethPrimer. A CpG island existed in the 2000 bp upstream sequences of the transcription start site of *TRPM1*. (B) IC_50_ of DNMT inhibitor 5-Aza-dC in D425 cells. (C) Expression level of *TRPM1* and miR-211 in D425 cells after 5-Aza-dC treatment was detected by qRT-PCR. (D) The effect of 5-Aza-dC on the proliferation of human MB cells by MTS assays. Data, mean ± SD. **P* < 0.05, ****P* < 0.001, NS, non-significant.


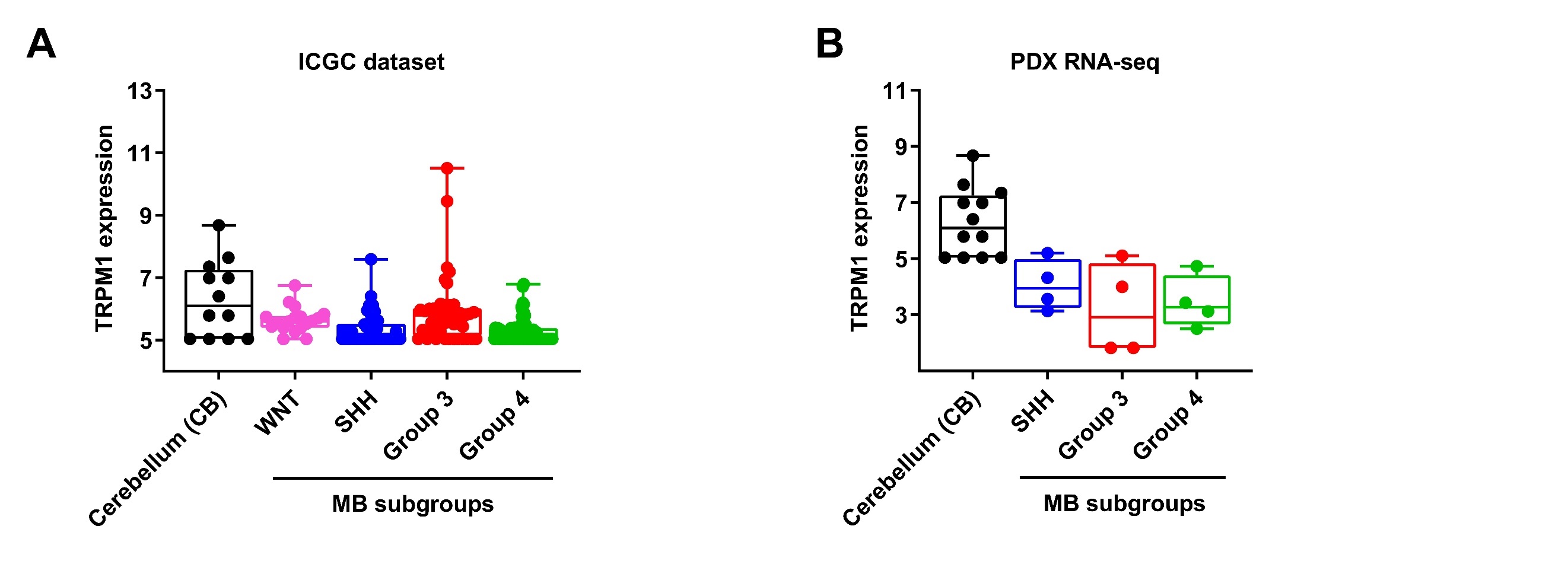


**Supplementary Figure 3. The expression of miR211's host gene *TRPM1* is downregulated in MB**.

(A) Boxplot showing distribution of normalized expression of *TRPM1* in the 4 molecular subgroups (WNT: 18, SHH: 46, Group 3: 45, Group 4: 66) of MB in the ICGC dataset (n = 175). (B) Boxplot distributions of expression levels of *TRPM1* in the 3 molecular subgroups (SHH: 4, Group 3: 4, Group 4: 4) of MB in the 12 PDX RNA-seq.


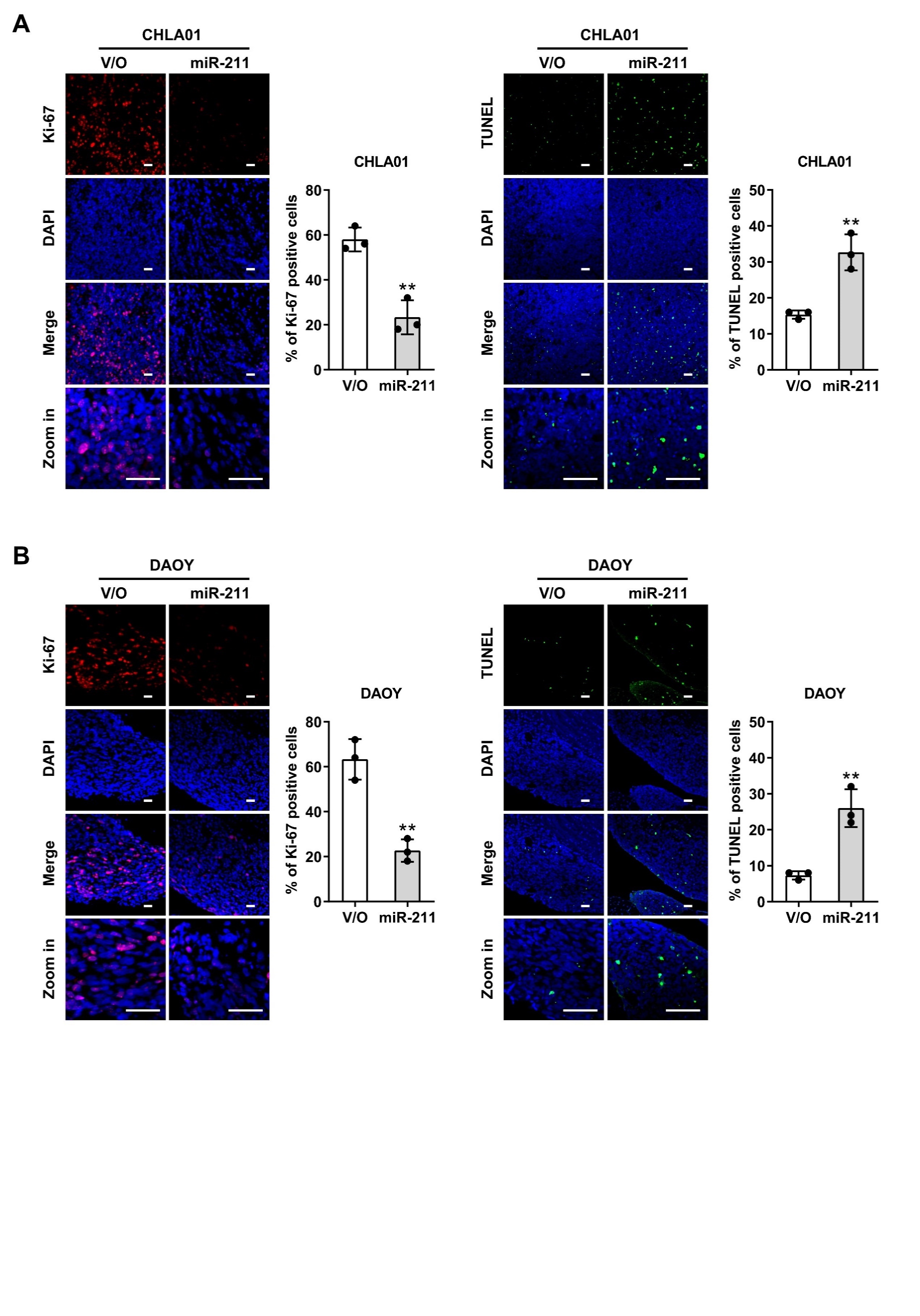


**Supplementary Figure 4. miR-211 inhibits the tumorigenicity of MB cells.**

(A) Fluorescent staining of Ki-67 and TUNEL in CHLA01 xenografts on day 42 after intracranial injection. Quantification of Ki-67 and TUNEL-positive cells is shown. (B) Fluorescent staining of Ki-67 and TUNEL in DAOY xenografts on day 35 after intracranial injection. Images are at a magniﬁcation of ×10 with a 10× zoom and at a magniﬁcation of ×40 with no zoom. Nuclei are stained with DAPI (blue). Scale bars, 100 μm. Data, mean ± SD. ***P* < 0.01.


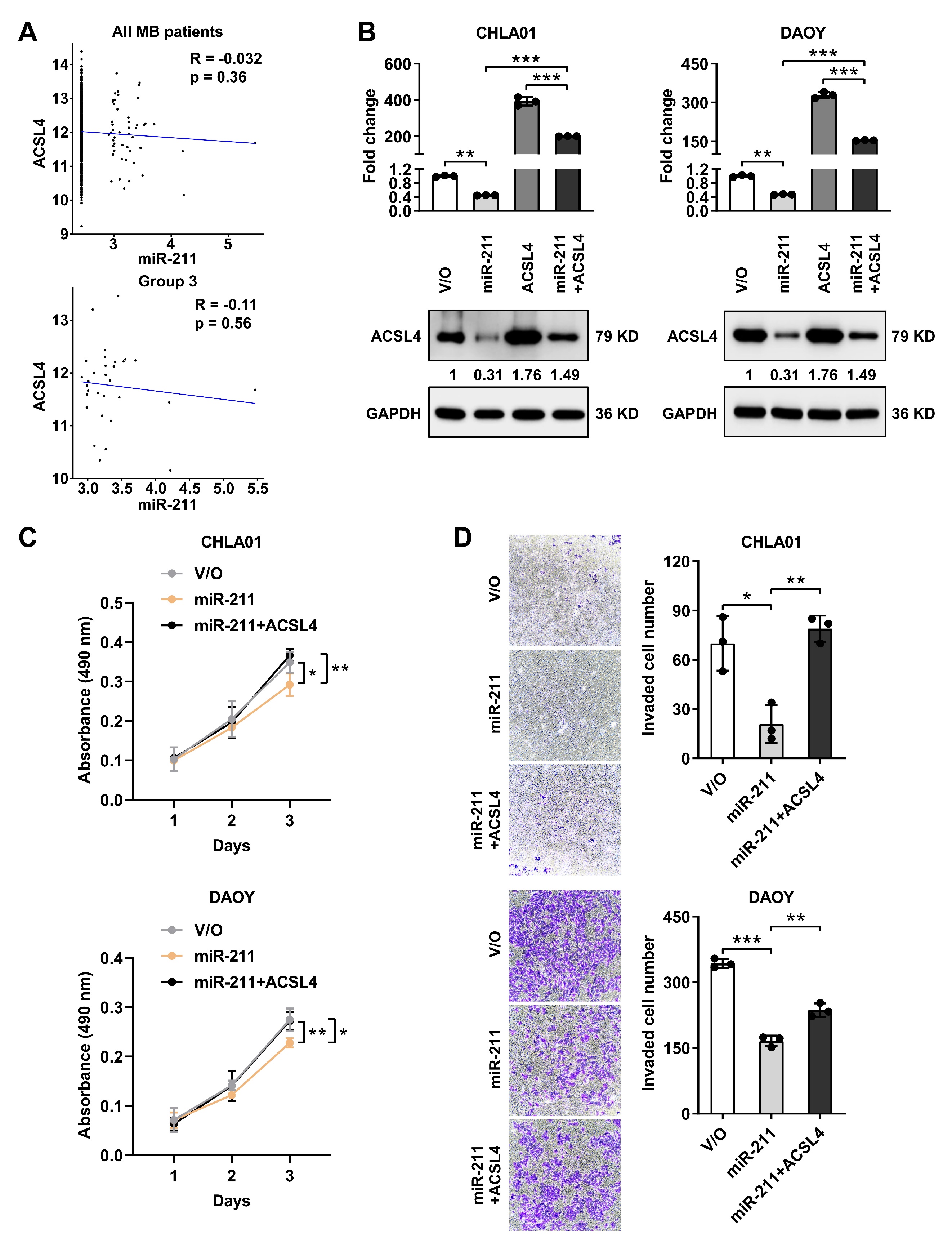


**Supplementary Figure 5. miR-211 inhibits MB progression by targeting *ACSL4* expression.**

(A) Correlation (expression levels) between miR-211 and *ACSL4* in MB patients from the MAGIC cohort. Upper panel all MB patients and the lower panel G3 MBs. (B) CHLA01 or DAOY cells expressing miR-211 or vector only (V/O) were transiently transfected with *ACSL4* plasmid, and lysates were applied to qRT-PCR and western blotting. (C) Cell proliferation was determined by MTS assays in CHLA01 or DAOY cells overexpressing miR-211 with and without introduction of *ACSL4* plasmid. (D) Cell invasive potential was determined by transwell assays in CHLA01 or DAOY cells overexpressing miR-211 with and without introduction of *ACSL4* plasmid. Data, mean ± SD. **P* < 0.05, ***P* < 0.01, ****P* < 0.001.


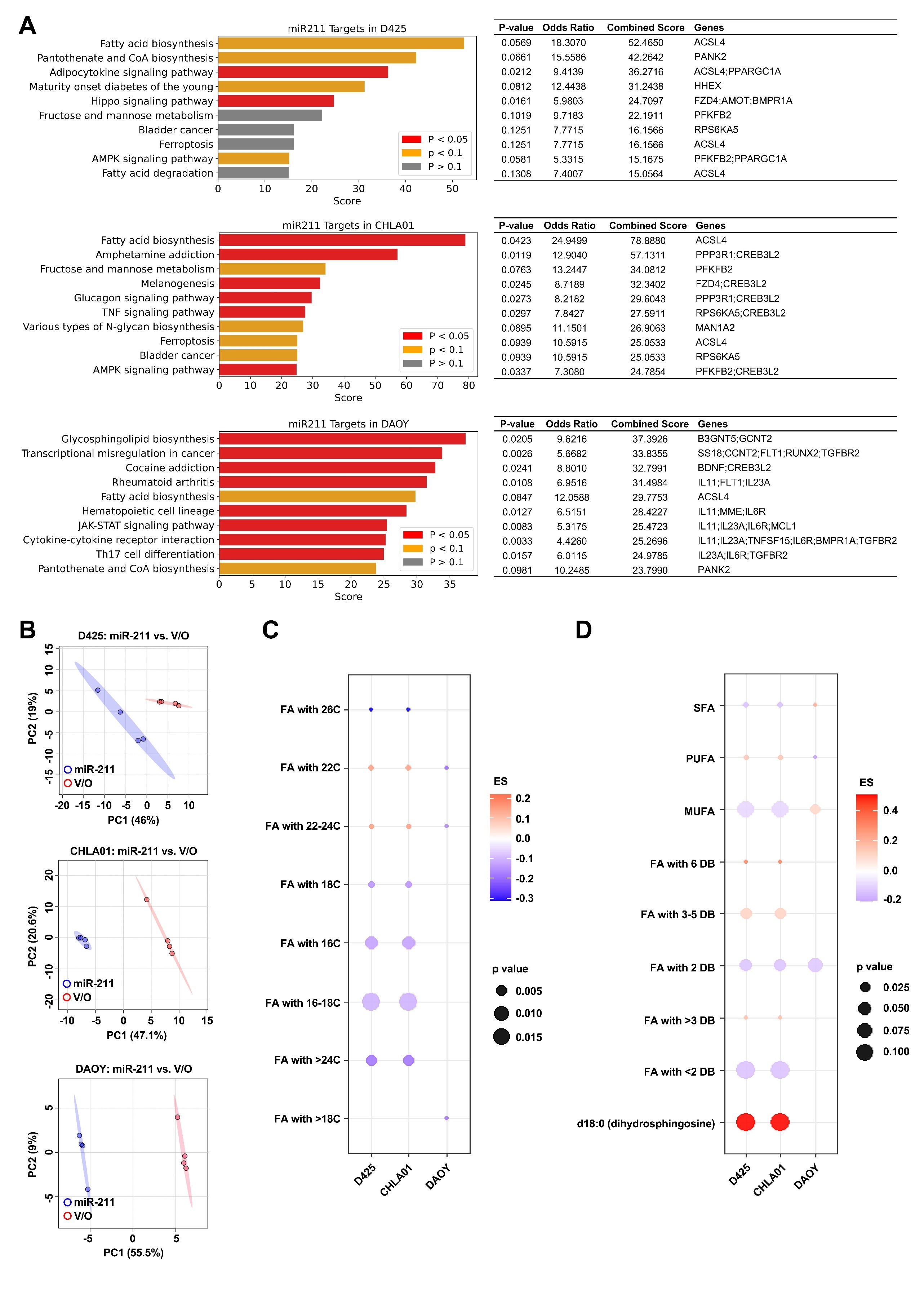


**Supplementary Figure 6. Lipidomic analysis of MB cells overexpressing miR-211.**

(A) D425, CHLA01 and DAOY cells expressing miR-211 are characterized by common metabolic process alterations according to KEGG pathway analysis using RNA-seq data. (B) Principal component analyses (PCA) in global lipidomic profiling of MB cells with or without miR-211 overexpression. (C) Fatty acid metabolism of different carbon chain lengths was detected upon miR-211 expression in MB cells. (D) The degree of lipid saturation was detected upon miR-211 expression in MB cells.


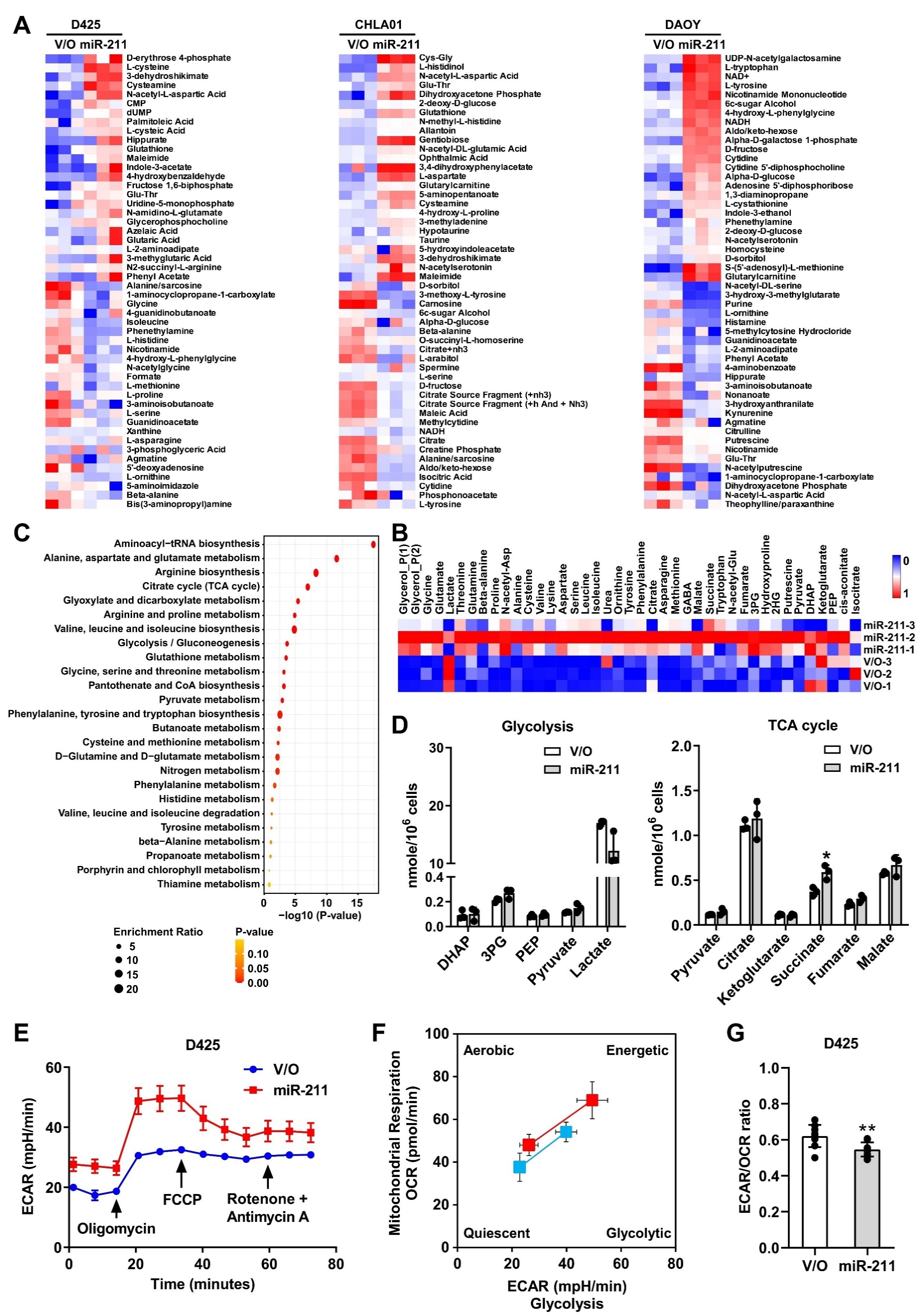


**Supplementary Figure 7. miR-211 is associated with a metabolic shift in MB cells.**

(A) Unsupervised hierarchical clustering analysis of D425, CHLA01 and DAOY cells expressing miR-211 global metabolome profiles. (B) Heat map of the 20 differential metabolites in D425 cells expressing vector only (V/O) or miR-211. (C) Pathway enrichment analysis related to the differential metabolites of D425. Color intensity (yellow to red) reflects increasing statistical significance. The graph was obtained by plotting on the x-axis the −log10 of p-values from pathway enrichment analysis and on the y-axis the pathway impact values derived from pathway topology analysis. (D) Quantitative analysis of intermediates for glycolysis and the tricarboxylic acid (TCA) cycle in D425 cells expressing miR-211 compared with control. (E) Extracellular acidiﬁcation rate (ECAR) was analyzed using the Seahorse XF analyzer in D425 cells expressing vector only (V/O) or miR-211. (F) Metabolic phenotype profiles in D425 cells representing changes in metabolic phenotype in response to miR-211 stress. (G) The basal ECAR/OCR ratios of D425 expressing V/O or miR-211 cells. Data, mean ± SD. **P* < 0.05, ***P* < 0.01.


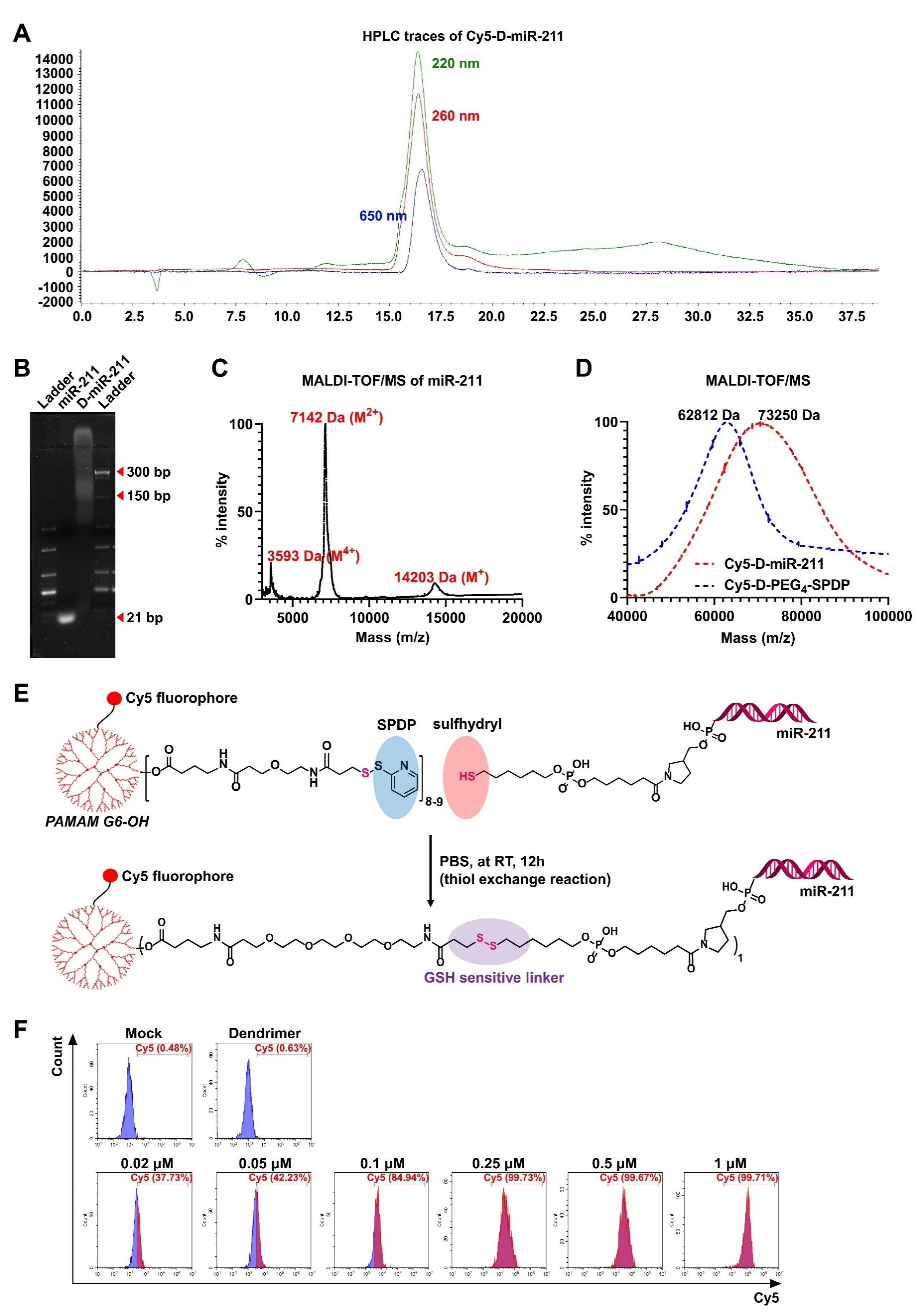


**Supplementary Figure 8. Synthesis and characterization of Cy5-Dendrimer-miR-211 and synthetic intermediates.**

(A) High-performance liquid chromatography (HPLC) traces of Cy5-Dendrimer-miR-211 at 220, 260, and 650 nm. (B) Gel electrophoresis of naked miR-211 and Cy5-Dendrimer-miR-211 on 10% TBE-Urea. The Cy5-Dendrimer-miR-211 band is retained around the 150 bp marker (~90 kDa). (C) Matrix-assisted laser desorption/ionization-time of flight (MALDI-TOF) mass spectrometry (MS) of miR-211. (D) MALDI-TOF/MS spectrum of Cy5-Dendrimer-miR-211. (E) Schematic of Dendrimer-miR-211 synthesis. The sulfhydryl group in 5ʹ-modified miR-211 was reacted (thiol exchange reaction in PBS at RT, 12 h) with Cy5-Dendrimer-PEG4-SPDP (upper panel) to obtain the final product Cy5-Dendrimer-miR-211 (lower panel). (F) D425 cells were treated with Cy5-Dendrimer-miR-211 conjugates starting from 0.02 μM to 1 μM for 24 h. Cy5 signaling was detected by flow cytometry Cy5 channel. Dendrimer without Cy5 labeling was used as a control.


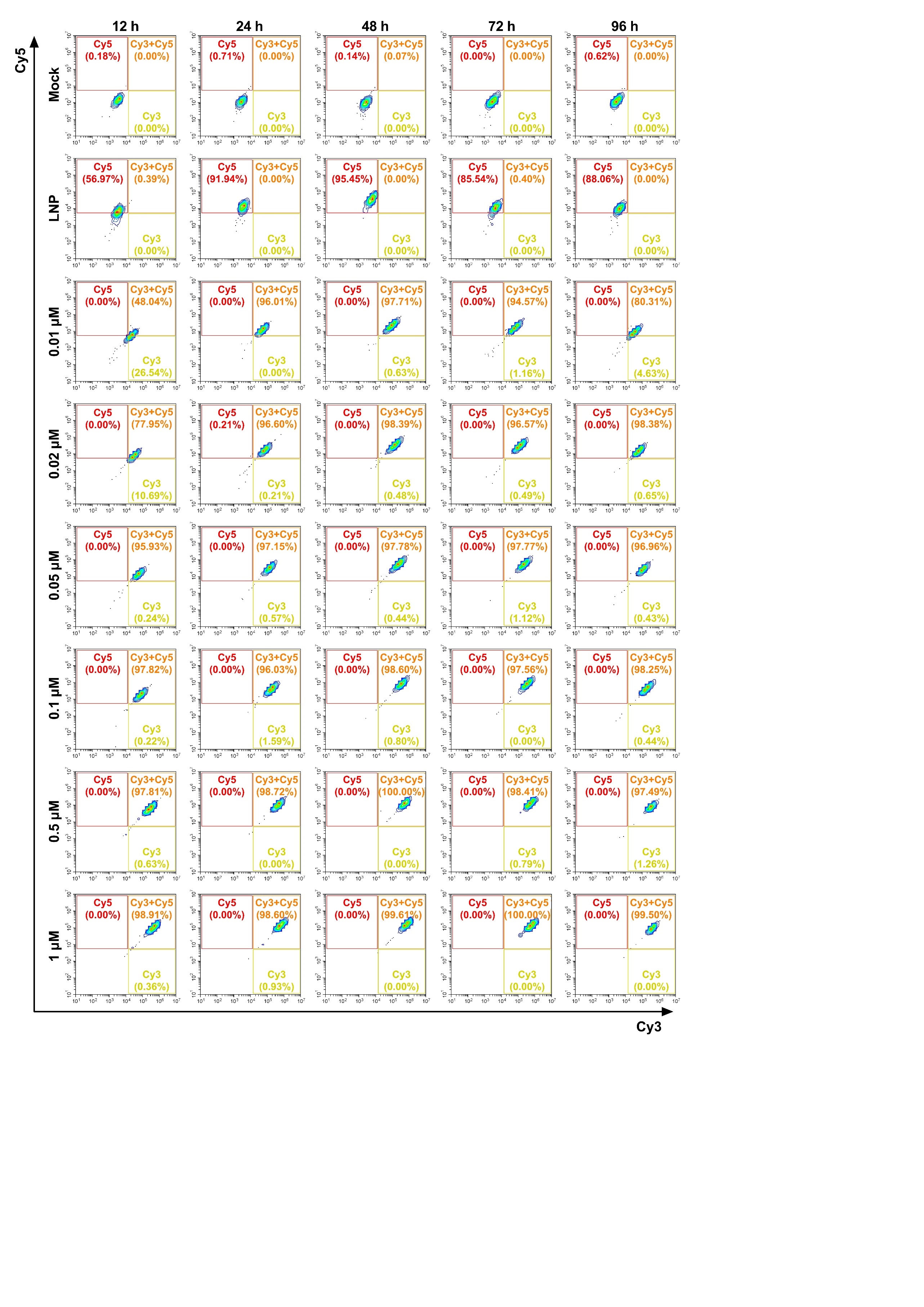


**Supplementary Figure 9. The intracellular uptake of lipid nanoparticles-miR-211 conjugates in MB cells.**

D425 cells were treated with LNP-miR-211 conjugates starting from 0.01 μM to 1 μM. Lipid nanoparticle was labeled with DiD dye and miR-211 was labeled with Cy3 dye. DiD and Cy3 signaling was detected by flow cytometry Cy5 and Cy3 channel, respectively, at different time point. LNP with DiD labeling was used as a control.


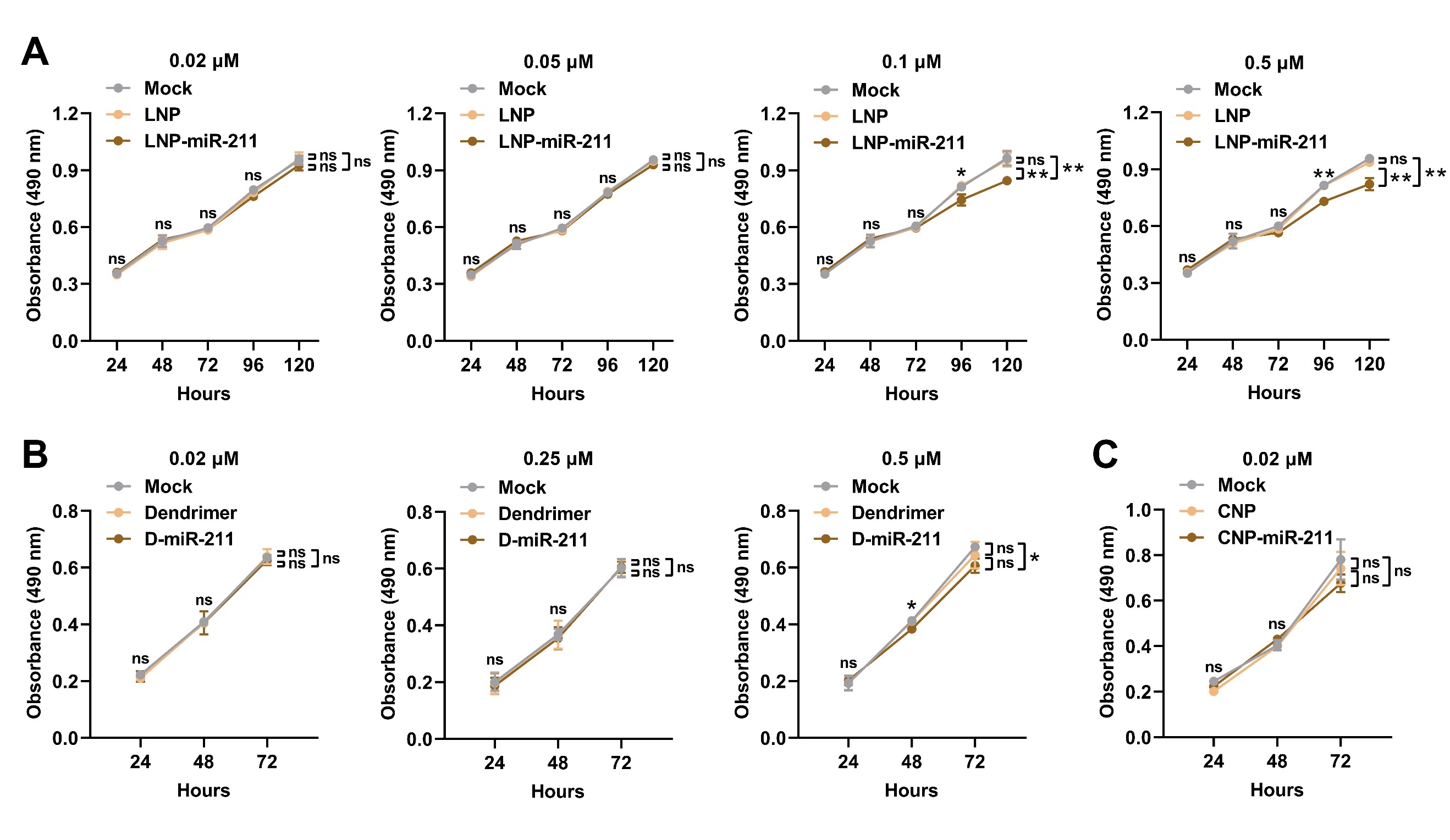


**Supplementary Figure 10. The effect of low concentrations of nanoparticle-miR-211 conjugates on MB cell proliferation.**

(A) The effect of 0.02, 0.05, 0.1, or 0.5 μM lipid nanoparticle-miR-211 (LNP-miR-211) on the proliferation of D425 cells by MTS assays. (B) The effect of 0.02, 0.25, or 0.5 μM Dendrimer-miR-211 (D-miR-211) on the proliferation of D425 cells by MTS assays. (C) The effect of 0.02 μM cerium oxide nanoparticle-miR-211 (CNP-miR-211) on the proliferation of D425 cells by MTS assays. Data, mean ± SD. ***P* < 0.01, NS, non-significant.

**SUPPLEMENTARY VIDEO**

**Supplementary video 1: The movie of AGO2/miR-211/*ACLS4* interaction.**

*ACLS4* 3′-UTR (orange) in complex with miR-211 (pink) and Argonaute2 (AGO2, white transparent). This 100-ns trajectory shows that *ACLS4* 3′-UTR strongly interacts with miR-211 bound to AGO2.
