# Supplementary material for: miRNA-211 maintains metabolic homeostasis in medulloblastoma through its target gene long-chain acyl-CoA synthetase 4
Source: Acta Neuropathol Commun. 2023 Dec 19;11:203. doi: 10.1186/s40478-023-01684-w (PMC10729563; doi:10.1186/s40478-023-01684-w)
Supplement: Supplementary file 2 — Additional file 2. Supplementary materials and methods. [file 40478_2023_1684_MOESM2_ESM.docx]

**Supplementary materials and methods**

**Cell transfection**

miR-211 mimics and mimic negative controls (miR-NC) were purchased from Dharmacon (Lafayette, CO). The plasmid containing *ACSL4* was obtained from Sino Biological (Beijing, China). We inoculated D425 cells into six-well plates at a 5 × 10^5^ cells/mL density and incubated them at 37°C with 5% CO_2_ for 24 h. All mimics were transfected at 20 nM or 2 µg of plasmids for 48 h using Lipofectamine 3000 (Invitrogen). Transfection efficiency was determined by quantitative real-time PCR (qPCR).

**Small RNA sequencing**

Small RNA was isolated and purified from MB cells using the miRNeasy Mini Kit (Qiagen, Hilden, Germany) following the manufacturer’s protocol and DNase digestion using the RNase-Free DNase Set (Qiagen). Purified RNA was eluted in 30 μl nuclease-free water (70°C) and quantified with the Qubit RNA HS. Libraries were constructed using the NEXTFLEX Small RNA Sequencing Kit V4 (PerkinElmer, Waltham, MA) following the manufacturer’s protocol. Size selection and cleanup of 145-160 nucleotide fragments were carried out using Cleanup Beads (PerkinElmer). The quality of DNA libraries was evaluated using the KAPA library qualification kit (Roche, Basel, Switzerland) and Agilent 4200 TapeStation (Agilent Technologies, Santa Clara, CA). Libraries were normalized to 2 nM and pooled libraries subjected to 75-nucleotide deep sequencing using the Illumina NextSeq 550 platform to obtain a minimum of 5 million reads per library. Small RNA-seq reads were checked for quality using FastQC. Low quality reads were trimmed using TrimGalore. Reads shorted than 16 base pairs were discarded from further analysis. Reads were aligned to the reference genome (hg38 gencode v42) using STAR aligner (v2.7.1a) with 10 maximum multiple mapping allowed. Read counts were obtained using the featureCounts tool using General Feature Format (gff) from miRBase database. Counts per million (CPM) normalization was performed using the edgeR package in R. Heatmaps and volcano plots were constructed using ComplexHeatMap and EnhancedVolcano packages in R.

**Chromogenic in situ hybridization (CISH)**

Chromogenic in situ hybridization (CISH) for hsa-miR-211-5p was carried out using miRNAscope HD Assay Red (ACD Bio, Newark, CA) according to the manufacturer's instructions. Four μm-thick formalin-fixed paraffin-embedded (FFPE) tissue sections of cerebellum and MB tissue microarrays were rehydrated, treated with hydrogen peroxide and target retrieval solution, followed by Protease III incubation. SR-has-miR-211-5p-S1 probe (ACD) was then added for 2 h at 40°C within the HybEZ Humidifying system. Signal amplification Amp1-6 was applied sequentially and incubated for 15 or 30 min. Fast red working solution was added for 10 min to detect the red signal. Samples were then counterstained with hematoxylin and mounted with EcoMount. Whole slide images were obtained at 40× using the Aperio VERSA system (Leica Microsystems, Wetzlar, Germany). Tissue sections were examined under a standard brightfield microscope at 40× magnification and scored semi-quantitatively according to the estimated number of punctate dots present within each cell boundary (score 0, no staining or less than 1 dot/cell; score 1, 2–10 dots/cell; score 2, 11–20 dots/cell, and score 3, >20 dots/cell).

**Ki-67 and TUNEL**

All 5 µm sections were dried for 30 min at 60°C. After removing paraffin and rehydrating the sections, antigen retrieval was performed by heating slides for 10 min with 0.1 M citrate buffer (pH 6.0). Tissue sections were blocked with 5% bovine serum albumin (BSA) and incubated with a rabbit anti-Ki-67 antibody (Abcam, ab15580) at 1:200 dilution in PBS for 1 h at room temperature. Then, goat anti-rabbit Alexa Fluor 546 (Invitrogen) was applied with DAPI, each incubated for 30 min at room temperature.

Apoptosis was quantified using the fluorescein terminal deoxynucleotidyl transferase (TdT) dUTP nick-end labeling assay using the deadEnd fluorometric TUNEL system (Promega). FFPE tissues sections were cut and rehydrated in gradient ethanol before permeabilization with proteinase K and covering with an equilibration buffer for 10 min at room temperature. Slides were incubated with TdT working solution at 37°C for 1 h and the reaction stopped with 2X SSC. Vectashield with DAPI counterstaining was applied to the slides, and fluorescence was detected by confocal fluorescence microscopy.

**Reporter assay**

TargetScan (https://www.targetscan.org/vert_80/) was used to predict possible miR-211 binding sites for *ACSL4*. The complete 3'-untranslated region (UTR) for human *ACSL4* was acquired by PCR from D425 genomic DNA as a template using Phusion high-fidelity DNA polymerase (New England Biolabs, Ipswich, MA). The *ACSL4* 3'-UTR sequence was cloned into pmirGLO dual-luciferase vector (Promega) between XbaI and PmeI sites, and miR-211 target site mutants were generated by site-directed mutagenesis using a Q5 site-directed mutagenesis kit (New England Biolabs). All *ACSL4* 3'-UTR wild (WT) and mutant type (MUT) vectors were confirmed by Sanger sequencing.

10^5^ D425 cells were seeded into 48-well plates and co-transfected with the pmirGLO vector carrying *ACSL4* 3'-UTR-WT or MUT with mimic negative controls or hsa-miR-211 mimics using Lipofectamine 3000 (Invitrogen), respectively. After 48 h of co-transfection, firefly and renilla luciferase activities were measured using the Dual-Glo luciferase assay (Promega) and the EnVision 2105 microplate reader (PerkinElmer).

**Untargeted lipidomics and metabolomics**

Cell samples were extracted using the modified Folch biphasic extraction procedure [1]. Briefly, for metabolomics, 20 μl of the standard internal mixture was added to cell pellets of 10 million cells. Protein was quantified for all samples for pre-normalization. Ice-cold methanol (80%) was used for global metabolome extraction. The supernatant was collected after centrifugation, transferred to a new tube, and dried under nitrogen. The dried sample was reconstituted in 0.1% formic acid in water (70 μl).

For lipidomics, 20 μl of 10× diluted standard internal mixture (stock solution of 50 ppm, w:v) was added. Samples were extracted using ice-cold 4:2:1 chloroform:methanol: water (v:v:v), and the organic phase was collected, dried under nitrogen flow, and reconstituted in 75 μl isopropanol plus 1 μl injection standard mixture (100 ppm, w:v) for global lipidome extraction. Metabolomics and lipidomics samples were run separately, and, for each sequence, solvent blanks, extraction blanks (without internal standards), neat quality controls (blanks with internal standards), and pooled sample quality controls were also prepared for evaluation of extraction and data collection efficiency. High-performance liquid chromatography and high-resolution tandem mass spectrometry (LC-HRMS/MS) were used for data collection. Chromatographic separation was achieved using reverse-phase chromatography (Thermo Scientific Dionex UltiMate 3000 RS UHLPC system; Thermo Fisher Scientific) with an Ace C18-PFP column (100 × 2.1 mm, 2 μmol/L) for metabolomics and with an Acquity UPLC BEH C18 column (Waters, Milford, MA) maintained at 30°C (2.1 × 100 mm, 1.7 μmol/L particle size) for lipidomics. In the case of metabolomics, the gradient consisted of solvent A (0.1% FA in H2O) and solvent B (acetonitrile), both with ten mmol/L ammonium formate and 0.1% formic acid. The flow rate was 350 μl/min. The column temperature was maintained at 25°C. In the case of lipidomics, the gradient consisted of solvent A (60:40 acetonitrile:water) and solvent B (90:8:2 isopropanol:aceto nitrile:water), both with 10 mmol/L ammonium formate and 0.1% formic acid. The flow rate was 500 μl/min. The column temperature was maintained at 50°C. Samples were analyzed in positive and negative electrospray ionization on a Thermo Scientific Q Exactive Orbitrap Mass Spectrometer (Thermo Fisher Scientific). Data-dependent (ddMS2-top5) MS/MS data were obtained on pooled samples per group for identification purposes. In addition, full-scan data were acquired for all the samples without MS/MS for comparing metabolite or lipid intensities across groups.

**MitoTracker Red CM-H2XRos assay**

We quantified mitochondria in live cells using the MitoTracker Red CM-H2XRos probe (Invitrogen). 10^5^ cells were spotted onto glass slides placed in 6-well plates and allowed to adhere for 2 h. Slides were covered with complete medium and incubated overnight with 5% CO_2_ at 37°C. Then, the intensity of red fluorescence produced by incubated cells was measured using 500 nM MitoTracker probe in the dark for 30 min. Slides were washed in PBS and incubated in Hoechst 33258 (2 μg/ml) for 10 min. Slides were scanned on a Nikon Ts2-FL fluorescence microscope (Nikon).

**Seahorse assay**

As per the manufacturer's instructions, mitochondrial activity of D425 cells was measured using the Seahorse Assay (Agilent). 1.5 × 10^4^ cells were plated in each well of a XFe96 cell culture microplate coated with poly-D-lysine (Sigma-Aldrich). OCR (oxygen consumption rate) and ECAR (extracellular acidification rate) were determined using an XFe96 extracellular flow analyzer every 7 min. After 15 min, we injected basal measurements with solutions of 5 μM oligomycin, 1 μM trifluoro carbonyl cyanide phenylhydrazone (FCCP), and then 0.5 μM rotenone-antimycin A. OCR and ECAR data were analyzed according to the Seahorse XFe96 protocol.

**Cell treatment with 5-Aza-dC**

D425 cells were seeded in 6-well plates and treated with 1, 5, 10, and 20 μM of the DNMT inhibitor 5-Aza-dC (decitabine, Selleckchem, Houston, TX) for 3 days. Medium containing drug was changed daily and cells were then harvested for RNA extraction.

**Cytotoxicity evaluation**

D425 cells were seeded in 96-well plates at a density of 5 × 10^3^ cells per well. 100 μl fresh medium containing 10 μl of 5-Aza-dC solutions at different concentrations were added to each well. Cells treated with DMSO were used as controls. After 72 h incubation, MTS solution (20 μl) was added to each well before incubating for another 3 h before quantification with an EnVision 2105 microplate reader (PerkinElmer) at a wavelength of 490 nm.

**LNP-miR-211 preparation and characterization**

An ionizable lipid LNP was prepared using microfluidic mixing. DLin-MC3-DMA, DSPC, cholesterol, and PEG-lipid were dissolved in ethanol at a molar ratio of 50:10:38.5:1.5 and 12.5 mM total lipid concentration. One volume of the lipid phase was mixed with 3 volumes of miR-211 dissolved in 50 mM citrate buffer pH 3 at 12 ml/min using the Ignite micromixer (Precision NanoSystems, Vancouver, BC, Canada). The nitrogen to phosphate (N/P) molar ratio of the amines in ionizable lipids to phosphate groups in miRNA was fixed at 3. The product was then diluted 40x in PBS and concentrated using 30 kD Amicon centrifugal filters (2000 × g, 4°C) to remove residual ethanol and exchange buffer. Double-stranded miR-211 was synthesized by Synbio Technologies (Monmouth Junction, NJ). For microscopic observation, miR-211 was conjugated with Cy3 and the LNP was labeled by adding DiD dye to the lipid phase. Size was measured using dynamic light scattering and zeta potential by laser Doppler electrophoresis. Encapsulation efficiency was determined using the Ribogreen assay.

**CNP-miR-211 synthesis and characterization**

The cerium oxide nanoparticles (CNPs) were synthesized using wet-chemical hydrolysis method at room temperature [2]. Five mM of cerium nitrate hexahydrate with a purity of 99.999% was dissolved in 48 ml of deionized water followed by the addition of 2 ml of hydrogen peroxide to the cerium solution. The solution was continuously mixed for 5 min. After the addition of hydrogen peroxide, the solution turned yellow and gradually became white after eight weeks of aging at room temperature. The fully aged nanoparticles were used for conjugation. For miR-211 conjugation with CNPs, initially, 270 µl of DMSO was taken in a 2 ml centrifuge tube [3], and then added 30 µl of CNPs (5 mM) [4]. The OH group on the CNPs surface was activated using 30 µl of CDI (500 mM) solution. After shaking the mixture for 1 h, 150 µl of miR-211 (200 µM) was added to the activated CNPs solution. The solution was mixed thoroughly by pipetting, and then 3790 µl of sodium borate buffer (10 mM, pH 8.5) was added. The solution was shaken at room temperature for 3 h. Following shaking, the solution was transferred into a 50 ml dialysis tube and dialyzed against RNase-free water at 4 °C for 20 h to remove the free miR-211 and DMSO solvent. The RNase-free water was replaced 2 h after starting dialysis. After dialysis, samples were collected and stored at -20 °C until further use. Following the manufacturer's protocol, the amount of miR-211 loaded on CNPs was quantified using the molecular probe Quant-iT microRNA assay kit (Invitrogen).

**Dendrimer-miR-211 synthesis and characterization**

Dendrimer-miR-211 conjugates were synthesized using a multistep reaction protocol established by the Kannan’s lab [5]. The PAMAM-G6-OH (D6-OH) dendrimer composed of ~256 terminal hydroxyl groups were used for this synthesis. The succinimidyl 3-(2-pyridyldithio) propionate (SPDP) linkers connected to near infrared-fluorescent tag Cyanine 5 (Cy5) attached dendrimer through amide bond, and miR-211-SH by Synbio technologies attached through glutathione-sensitive disulfide bond through classical thiol-disulfide interchange by miR-211-SH. The sense 5ʹ disulfide modified miR-211 duplex was used for this study. Prior to use, the thiol-modified (S-S) miR-211 was reduced to sulfhydryl (-SH) for further conjugation. The dithiol modified miR-211 was treated with 100 mM of dithiothreitol (DTT) to quantitatively reduce disulfide bonds, resulting in sulfhydryl groups for further conjugation with dendrimer. The resulting sulfhydryl group in the sense 5ʹ end of miR-211 was then reacted with Cy5-D-PEG4-SPDP to form desired Cy5-D-miR-211 conjugate via a thiol exchange reaction. The resulted Cy5-D-miR-211 was passed through GE Healthcare Sephadex G-25 column and concentrated by ultrafiltration. The purity of the product was evaluated using high-performance liquid chromatography (HPLC) and the molecular weight was determined by Matrix assisted laser desorption microscopy (MALDI-TOF). The successful synthesis of Cy5-D-miR-211 was confirmed by the presence of a band from Cy5-D-miR-211 at a distance corresponding 150 bp marker on the TBE-Urea gel, with an estimated size of 90 kDa in size (Supplementary Fig 8).

**Dendrimer-miR-211 synthesis**

Unless stated otherwise, reactions were performed in flame-dried glassware under a positive pressure of N2. Commercial grade reagents and solvents were used without further purification except where noted. Proton nuclear magnetic resonance (^1^H NMR) spectra were recorded on a Bruker 500 MHz spectrometer at ambient temperatures and analyzed using Mnova software. ^1^H NMR chemical shifts were reported as δ using residual solvent as an internal standard (DMSO-*d6*, 2.50), and (D_2_O, 4.79 ppm). Cyanine 5 (Cy5)-mono-NHS ester was purchased from Amersham Bioscience-GE Healthcare. Deuterated solvents dimethylsulfoxide (DMSO-*d6*), water (D_2_O), and Chloroform (CDCl_3_) were purchased from Cambridge Isotope Laboratories Inc. (Andover, MA). Ethylenediamine-core polyamidoamine (PAMAM) dendrimer, generation 6.0, hydroxy surface (G6-OH; diagnostic grade; consisting of 256 hydroxyl end-groups), methanol solution (13.75% w/w) was purchased from Dendritech Inc. (Midland, MI, USA). Dialysis membranes were purchased from Spectrum Laboratories Inc. (Rancho Dominguez, CA, USA).

**Synthesis of Cy5-Dendrimer-PEG4-SPDP**

A solution of Cy5-Dendrimer, (125 mg, 0.002 mmol) in DMF (2 ml) was treated with DIPEA to adjust pH of the reaction mixture (~7.0-7.5). The reaction was treated with SPDP-PEG4-NHS ester (6 mg, 0.0010 mmol) and stirred the reaction mixture at room temperature for 12 h. It was then dialyzed against DMF 12 h followed by against water for 24 h. The aqueous layer was frozen and lyophilized to yield desired product, 1 as a blue solid (yield 80%). ^1^H NMR (500 MHz, DMSO-*d6*) δ 8.25-7.75 (m, internal amide H), 7.35 (m, Cy5 H), 7.25 (m, Cy5 H), 7.05 (m, Cy5 H), 6.6 (m, Cy5 H), 6.3 (m, Cy5 H), 6.83 (s, GABA amide H), 4.74 (s, surface OH, H), 4.01-3.39 (t, J = 5.0 Hz, ester –CH_2_), 3.50-2.00 (m, dendrimer CH_2_), 1.9 (s, 24H), 1.6 (s, 80H), 1.2 (s, 126H), 0.8 (s, 80H). HPLC C18 retention time: 19.5 min.

**Synthesis of Dendrimer-miR-211 conjugates**

Thiol-modified miR-211 was dissolved in 125 µl of DTT solution (100 mM solution of DTT in 100 mM sodium phosphate buffer, pH 8.3-8.5 was prepared by dissolving 77.13 mg of DTT in 5 ml buffer) and incubated at room temperature for 1 hr. Byproduct removal was done using GE Healthcare NAP-10 columns Sephadex G-25 DNA grade (CAS No. 2682-20-4). The thiol-modified miR-211 was eluted to Amicon Ultra-centrifuge, Ultracel 10K filters (UFC501024) with 0.5 ml sodium phosphate buffer. A solution of dendrimer (6.97 mg, 116.17 nmol, 1.0 eq) in 200 µl was treated with miR-211-SH (69.7 nmol, 1.2 eq) in 200 µl and stirred the reaction mixture at room temperature. After 12 h the mixture was passed through a GE Healthcare Sephadex G-25 column and Cy5-Dendrimer-miR-211 product was collected. The product was concentrated and buffer exchange to PBS by centrifuged ultra-filtration using a 0.5 ml capacity 30KDa MWCO filter unit.

**Ultrafiltration and SEC chromatography**

The buffer exchange, removal of excess of reagents and byproducts were performed by ultracentrifugal filtration using 0.5 ml Amicon filtration units with MWCO 30 kDa or 100 kDa. The Products and intermediated were further purified by Size-exclusion column (SEC) chromatography using PBS as the mobile phase.

**Characterization of Cy5-Dendrimer-miR-211 conjugates using analytical high-performance liquid chromatography (HPLC)**

The purity of Cy5-Dendrimer-miR-211 was analyzed using HPLC (Shimadzu LC-AD HPLC system). The instrument is equipped with a variable wavelength absorbance detector and a C18 reverse phase column (Waters, BEH300 5 µm, 19×250 mm). The eluents were monitored at 210 nm, 260 nm and 650 nm using a photodiode array (PDA) detector. HPLC elution was carried out with a 40 min linear gradient of 0%-90% HPLC grade acetonitrile (CH_3_CN) in water (containing 0.1% TFA) maintaining the flow rate at 1.0 ml/min.

**Sample preparation and MALDI-TOF analysis**

The MALDI-TOF MS analysis was performed on a Bruker Voyager DE-STR MALDI-TOF (Mass Spectrometric and Proteomics core, Johns Hopkins University, School of Medicine) operated in linear, positive ion mode.

**PAMAM dendrimer intermediates and conjugates**

The MALDI matrix 2ʹ,4ʹ,6ʹ-Trihydroxyacetophenone monohydrate (THAP) (10 mg in 1ml of Acetonitrile in water (1:1) with 0.1% trifluoroacetic acid) was used for analysis of dendrimer intermediates and conjugates. The PAMAM dendrimer/dendrimer conjugates (2 µl) were deposited on the MALDI sample plate. The matrix (2 µl of the 10 mg/ml) was deposited on the air-dried sample and allowed it to air dry for 10-20 min. The MALDI-TOF MS analysis was performed in a reflective-positive mode.

**Oligonucleotides**

Matrix containing 3-hydroxypicolinic acid (3-HPA), and di-ammonium hydrogen citrate (DAHC) was used for oligonucleotide analysis. A solution of 3-HPA (50 mg/ml in 50% MeCN/water) was mixed with DAHC solution (100 mg/ml in 50% MeCN/water) in 9:1 ratio (225 µl of 3-HPA: 25 µl DAHC) to give final DAHC concentration 10 mg/ml. miR-211 solution was desalted prior to mixing with matrix and 2 µl of miR-211 was deposited on the plate and allowed it to air dry for 10-20 min. Then HPA/DAHC matrix (2 µl) was deposited on the air-dried oligonucleotide and allowed it to air dry.

**Gel electrophoresis**

A gel retardation assay was performed to confirm the formation of the Cy5-Dendrimer-miR-211 conjugates. RNA ladder (NEB, Ipswich, MA), free miR-211, and Cy5-Dendrimer-miR-211 were mixed with GelRed stain, 1 µl of glycerol, and ultrapure water for a nucleic acid loading of 2 µg. Gel electrophoresis was performed in 10% TBE-Urea gel with TBE buffer (Bio-Rad, Hercules, CA) at 120 V for 20 min, after which the gel was imaged in a ChemiDoc Imaging System (Bio-Rad, Hercules, CA).

**References**

1 Lees M, Folch J, Stanley GH, Carr S (1959) A simple procedure for the preparation of brain sulphatides. J Neurochem 4: 9-18 Doi 10.1111/j.1471-4159.1959.tb13169.x

2 Neal CJ, Sakthivel TS, Fu Y, Seal S (2021) Aging of Nanoscale Cerium Oxide in a Peroxide Environment: Its Influence on the Redox, Surface, and Dispersion Character. The Journal of Physical Chemistry C 125: 27323-27334 Doi 10.1021/acs.jpcc.1c06279

3 El Ghzaoui C, Neal CJ, Kolanthai E, Fu Y, Kumar U, Hu J, Zgheib C, Liechty KW, Seal S (2022) Assessing the bio-stability of microRNA-146a conjugated nanoparticles via electroanalysis. Nanoscale Adv 5: 191-207 Doi 10.1039/d2na00600f

4 Fu Y, Kolanthai E, Neal CJ, Kumar U, Zgheib C, Liechty KW, Seal S (2022) Engineered Faceted Cerium Oxide Nanoparticles for Therapeutic miRNA Delivery. Nanomaterials (Basel) 12: Doi 10.3390/nano12244389

5 Liyanage W, Wu T, Kannan S, Kannan RM (2022) Dendrimer-siRNA Conjugates for Targeted Intracellular Delivery in Glioblastoma Animal Models. ACS Appl Mater Interfaces 14: 46290-46303 Doi 10.1021/acsami.2c13129
